# Genetic Interaction between MTMR2 and FIG4 Phospholipid Phosphatases Involved in Charcot-Marie-Tooth Neuropathies

**DOI:** 10.1371/journal.pgen.1002319

**Published:** 2011-10-20

**Authors:** Ilaria Vaccari, Giorgia Dina, Hélène Tronchère, Emily Kaufman, Gaëtan Chicanne, Federica Cerri, Lawrence Wrabetz, Bernard Payrastre, Angelo Quattrini, Lois S. Weisman, Miriam H. Meisler, Alessandra Bolino

**Affiliations:** 1 Human Inherited Neuropathies Unit, INSPE-Institute for Experimental Neurology, Division of Neuroscience, San Raffaele Scientific Institute, Milan, Italy; 2 Dulbecco Telethon Institute, San Raffaele Scientific Institute, Milan, Italy; 3 Neuropathology Unit, INSPE–Institute for Experimental Neurology, Division of Neuroscience, San Raffaele Scientific Institute, Milan, Italy; 4 INSERM U1048 and Université Toulouse 3, I2MC, CHU Toulouse, Toulouse, France; 5 Biology of Myelin Unit, Division of Genetics and Cell Biology, San Raffaele Scientific Institute, Milan, Italy; 6 Life Science Institute, University of Michigan, Ann Arbor, Michigan, United States of America; 7 Department of Human Genetics, University of Michigan, Ann Arbor, Michigan, United States of America; The Jackson Laboratory, United States of America

## Abstract

We previously reported that autosomal recessive demyelinating Charcot-Marie-Tooth (CMT) type 4B1 neuropathy with myelin outfoldings is caused by loss of MTMR2 (Myotubularin-related 2) in humans, and we created a faithful mouse model of the disease. MTMR2 dephosphorylates both PtdIns3*P* and PtdIns(3,5)*P*_2_, thereby regulating membrane trafficking. However, the function of MTMR2 and the role of the MTMR2 phospholipid phosphatase activity *in vivo* in the nerve still remain to be assessed. Mutations in *FIG4* are associated with CMT4J neuropathy characterized by both axonal and myelin damage in peripheral nerve. Loss of Fig4 function in the *plt* (pale tremor) mouse produces spongiform degeneration of the brain and peripheral neuropathy. Since FIG4 has a role in generation of PtdIns(3,5)*P*_2_ and MTMR2 catalyzes its dephosphorylation, these two phosphatases might be expected to have opposite effects in the control of PtdIns(3,5)*P*_2_ homeostasis and their mutations might have compensatory effects *in vivo*. To explore the role of the MTMR2 phospholipid phosphatase activity *in vivo*, we generated and characterized the Mtmr2/Fig4 double null mutant mice. Here we provide strong evidence that Mtmr2 and Fig4 functionally interact in both Schwann cells and neurons, and we reveal for the first time a role of Mtmr2 in neurons *in vivo*. Our results also suggest that imbalance of PtdIns(3,5)*P*_2_ is at the basis of altered longitudinal myelin growth and of myelin outfolding formation. Reduction of *Fig4* by null heterozygosity and downregulation of PIKfyve both rescue *Mtmr2*-null myelin outfoldings *in vivo* and *in vitro*.

## Author Summary

Charcot-Marie-Tooth type 4B1 (CMT4B1) and Charcot-Marie-Tooth type 4J (CMT4J) are severe autosomal recessive demyelinating neuropathies with childhood onset. We previously demonstrated that loss of the phospholipid phosphatase MTMR2 causes CMT4B1 with myelin outfoldings in human and mouse and that loss of the phospholipid phosphatase FIG4 causes CMT4J and neurodegeneration in the mouse. MTMR2 has a predicted role in membrane trafficking, which is crucial for myelin membrane biogenesis and homeostasis. However, the biochemical activity of MTMR2 *in vivo* and the role of MTMR2 in myelination still remain to be assessed. MTMR2 and FIG4 act on the same phospholipid substrate PtdIns(3,5)*P*_2_, but with predicted opposite effects. We generated a double *Mtmr2*/*Fig4*-null mouse which showed that Mtmr2 and Fig4 interact in neurons and Schwann cells to control phospholipid metabolism. Moreover, *Mtmr2*-null myelin outfoldings are rescued by *Fig4* heterozygosity, suggesting that imbalance of PtdIns(3,5)*P*_2_ is at the basis of the excessive myelin growth and hypermyelination.

## Introduction

Phosphoinositides (PIs) constitute potent signaling molecules with a specific and restricted distribution at intracellular membranes that is strictly controlled by the concerted action of kinases and phosphatases [1], [2]. PIs are key regulators of membrane trafficking as they contribute to assembly of molecular machineries that promote and control membrane dynamics and vesicle movement, tethering and fusion. In the nervous system, both neurons and glia rely on efficient membrane trafficking for many functions, such as axonal transport or myelination.

Charcot-Marie-Tooth (CMT) neuropathies are very heterogeneous disorders from both the clinical and genetic point of view [3]–[6]. Several CMT genes encode proteins that regulate or are connected with PI metabolism, including FRABIN/FGD4, FIG4, DNM2, RAB7, SIMPLE, LRSAM1, SH3TC2, MTMR2, and MTMR13, supporting the idea that regulation of intracellular trafficking is a key process in peripheral nervous system biology [7] (http://www.molgen.ua.ac.be/CMTMutations/default.cfm).

We first demonstrated that loss of function mutations in the *MTMR2* (Myotubularin-related 2) gene cause autosomal recessive demyelinating Charcot-Marie-Tooth type 4B1 (CMT4B1, OMIM #601382) neuropathy with myelin outfoldings [8]. MTMR2 is a phospholipid phosphatase that dephosphorylates both PtdIns3*P* and PtdIns(3,5)*P*_2_ phosphoinositides at the D3 position of the inositol ring, thus generating PtdIns5*P* [9]–[16]. We have generated a *Mtmr2*-null mouse which models the CMT4B1 neuropathy and we reported that loss of Mtmr2 specifically in Schwann cells is both sufficient and necessary to provoke myelin outfoldings [17], [18]. We recently identified a potential mechanism using *in vivo* and *in vitro* models of CMT4B1 and proposed that Mtmr2 belongs to a molecular machinery that titrates membrane formation during myelination. According to this model, myelin outfoldings arise as a consequence of the loss of negative control on the amount of membrane produced during myelination [19]. Despite these findings, the function of MTMR2 and the role of the MTMR2 phospholipid phosphatase activity in the nerve still remain to be assessed.

Loss of the FIG4/SAC3 phospholipid phosphatase in human provokes another form of autosomal recessive demyelinating CMT, the CMT type 4J (OMIM #611228) neuropathy [20], [21]. FIG4 is a 5-phosphatase involved in the dephosphorylation of PtdIns(3,5)*P*_2_, a predicted substrate of MTMR2. Loss of Fig4 in the mouse causes the *plt* (pale tremor) phenotype, characterized by extensive neuronal vacuolization and degeneration and by a peripheral neuropathy [20], .

Yeast Fig4 is localized at the vacuolar membrane-the yeast lysosomal compartment- and is required for both the generation and turnover of PtdIns(3,5)*P*_2_ [23], [24]. In addition to the 5-phosphatase activity, yeast Fig4 appears to activate Fab1, the kinase that produces PtdIns(3,5)*P*_2_ from PtdIns3*P* [23], [24]. Deletion of yeast Fig4 reduces rather than increases PtdIns(3,5)*P*_2_ leading to defects in vacuole homeostasis and function. A significant decrease of PtdIns(3,5)*P*_2_ was found also in *plt* (*Fig4*-null) fibroblasts, suggesting conserved enzymatic and cellular functions of Fig4 from yeast to mouse [20]. Moreover, the most common human mutation of *FIG4* acts by reducing its affinity for the PtdIns(3,5)*P*_2_ biosynthetic complex [25].

Since FIG4 has a role in generation of PtdIns(3,5)*P*_2_ and MTMR2 catalyzes its dephosphorylation, these two phosphatases might have opposite effects in the control of PtdIns(3,5)*P*_2_ homeostasis and their mutations might have compensatory effects *in vivo*. To explore the role of the MTMR2 phospholipid phosphatase activity *in vivo*, we took advantage of the *Fig4* and *Mtmr2*-null mice and generated and characterized the Mtmr2/Fig4 double null mutant. Here we provide strong evidence that Mtmr2 and Fig4 functionally interact in both Schwann cells and neurons, and reveal for the first time a role of Mtmr2 in neurons *in vivo*. We also report that the imbalance of PtdIns(3,5)*P*_2_ might be at the basis of myelin outfolding in the nerve. Reduction of *Fig4* by null heterozygosity and downregulation of PIKfyve both rescue *Mtmr2*-null myelin outfoldings *in vivo* and *in vitro*.

## Results

### Generation of *Mtmr2/Fig4*-null mice

The generation and characterization of *Mtmr2*-null and *Fig4*-null (*plt*) mice have been reported [17], [20]. *Mtmr2/Fig4* double null mice and controls were analyzed in the F2 generation. At postnatal day three (P3) *Mtmr2−/−Fig4−/−* mice had reduced body size and diluted pigmentation of the coat similar to the *Mtmr2+/+Fig4−/−* mice in the same litter, and as reported for the *plt* mouse [20]. Tremor and abnormal gait developed in the second week after birth. *Mtmr2+/+Fig4−/−* mice show juvenile lethality and die around 1 month of age. The viability of *Mtmr2−/−Fig4−/−* mice was lower than for *Mtmr2+/+Fig4−/−* littermates. A reduced number of both *Mtmr2*+/−*Fig4*−/− and *Mtmr2−/−Fig4−/−* mice were present at P8, compared to the expected Mendelian ratio (Table 1 and Table 2). The longest survival of the double mutant was 20 days. The data indicate that loss of Mtmr2 reduces viability of *Mtmr2+/+Fig4−/−*. We therefore hypothesized that loss of Mtmr2 might provoke a worsening of the *Mtmr2+/+Fig4−/−* neurodegeneration.

**Table 1:** Number of mice with *Mtmr2*+/+*Fig4*−/−, *Mtmr2*+/−*Fig4*, and *Mtmr2*−/−*Fig4*−/− genotypes scored at P8 on a total of 608 mice.

MTMR2	FIG4	Predicted	Observed	P values
+/+	−/−	38	34	0.28
+/−	−/−	76	56	0.007
−/−	−/−	38	10	<0.0001

“Predicted” refers to the number of expected mice among 608 mice born, based on Mendelian ratios. “Observed” refers to the mice identified by genotyping at P8. The number of mice decreases with loss of Mtmr2 on a *Fig4*-null background. P values were calculated for a binomial distribution, using the chi-square test.

**Table 2:** The relative risk (RR) of lethality for the *Mtmr2*+/+*Fig4*−/−, *Mtmr2*+/−*Fig4*−/−, and *Mtmr2*−/−*Fig4*−/ genotypes.

Genotype 1	Genotype 2	RR (95%CI)	Chi-square	P values
*Mtmr2*+/+*Fig4*−/−	*Mtmr2*+/−*Fig4*−/−	2.5 (0.92, 6.80)	2.91	0.09
*Mtmr2*+/+*Fig4*−/−	*Mtmr2*−/−*Fig4*−/−	7 (2.72, 18.03)	28.55	9×10^−8^
*Mtmr2*+/−*Fig4*−/−	*Mtmr2*−/−*Fig4*−/−	2.8 (1.84, 4.27)	21.42	4×10^−6^

The relative risk analysis was performed on the 608 animals scored at P8, as also reported in Table 1.

### Mtmr2 loss exacerbates *Fig4*-null neurodegeneration

**Figure 1:**
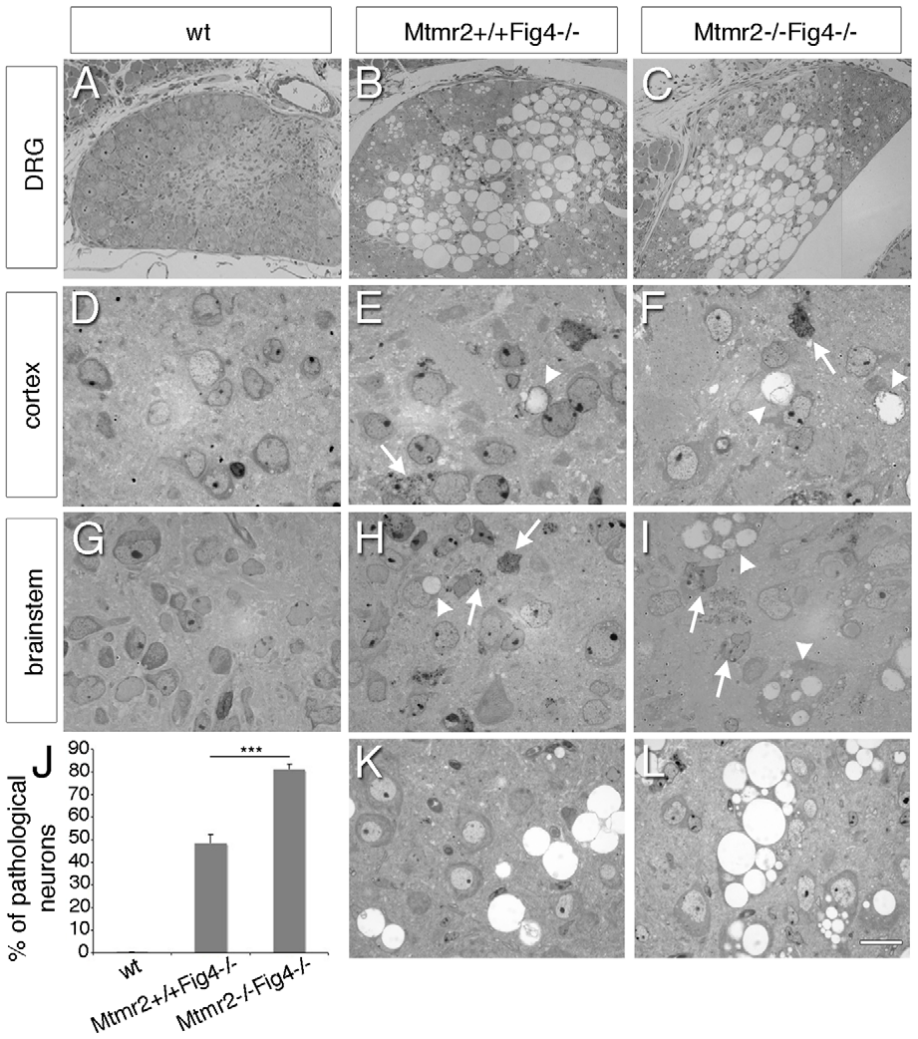
Neurodegeneration in *Mtmr2*+/+*Fig4*−/− and *Mtmr2*−/−*Fig4*−/− mice. Semithin section analysis of DRG ganglia, cortex and brainstem of wild-type (A, D, G) and mutant mice (B, C, E, F, H, I). (B, C) DRG sensory neurons from *Fig4*-null and *Mtmr2*/*Fig4* double null mice at P3 with massive vacuolization. (F, I) More cells carrying inclusions and vacuoles were observed in *Mtmr2*−/−*Fig4*−/− cortex (F) and brainstem (I) at P3 as compared to *Mtmr2*+/+*Fig4*−/− mice (E, H). (K, L) Brainstem analysis at P8 showed increased number of pathological neurons in *Mtmr2*−/−*Fig4*−/− (L) as compared to *Mtmr2*+/+*Fig4*−/− mice (K) and quantification in (J), P = 2.75×10^−6^. A total of 10030, 10290, and 9620 neurons were counted and analyzed in wild-type *Mtmr2*+/+*Fig4*−/−, and *Mtmr2*−/−*Fig4*−/− mice, respectively. All neurons scored were normal in wild-type, whereas 48.3% and 81.1% of neurons displayed pathological features in *Mtmr2*+/+*Fig4*−/−, and *Mtmr2*−/−*Fig4*−/−, respectively. Arrows indicate inclusions; arrowheads vacuoles. Three mice per genotype were analyzed. Bar is 35 µm in (A–C), 10 µm in (D–L).

To explore this possibility, we performed semithin section analysis of DRG ganglia, brain and spinal cord from *Mtmr2+/+Fig4−/−* and *Mtmr2−/−Fig4−/−* mice. DRG ganglia from both *Mtmr2*+/+*Fig4*−/− and *Mtmr2*−/−*Fig4*−/− mice at P3 were severely affected, exhibiting neuronal loss and massive vacuolization (Figure 1A–1C). In the cerebellum of both *Mtmr2*+/+*Fig4*−/− and *Mtmr2*−/−*Fig4*−/− mice at P8 and at P20 we observed a thickening of the molecular layer as compared to wild-type, and cells with cytoplasmic vacuoles were present in the granular layer. At P20, a consistent loss of Purkinjie and basket cells was observed in both genotypes (Figures S1 and S2). These cerebellar findings have not been previously reported in the *plt* mouse.

**Figure 2:**
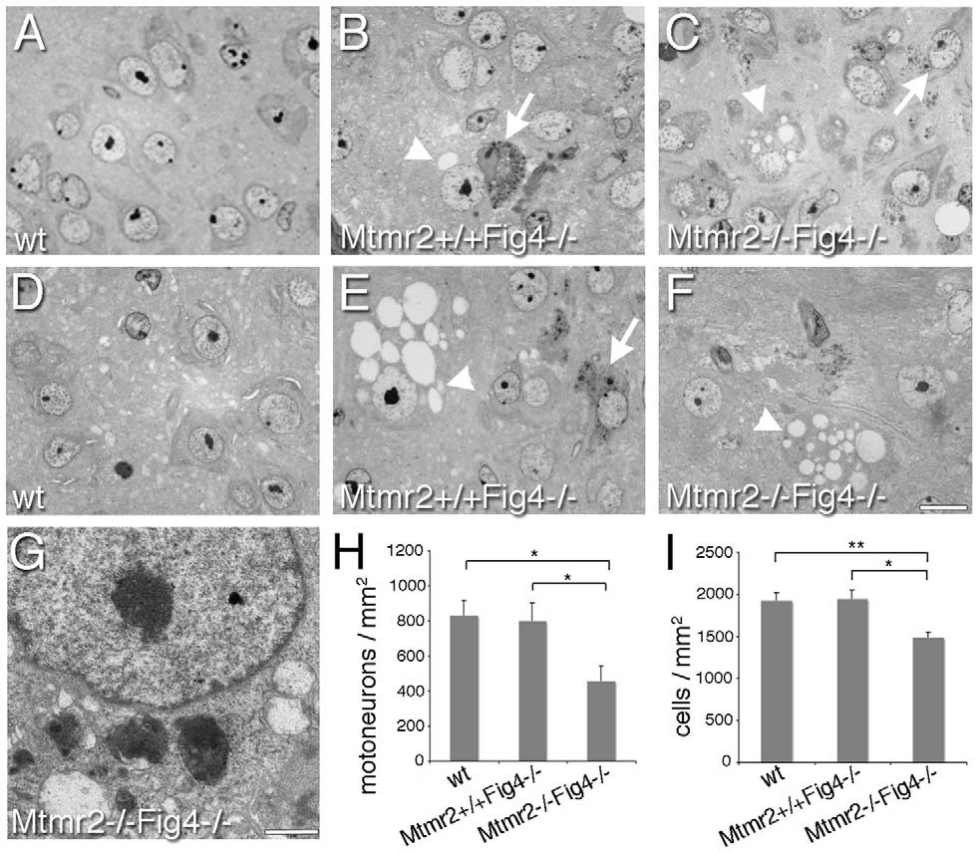
Semithin section analysis of spinal cord from *Mtmr2*+/+*Fig4*−/− and *Mtmr2*−/−*Fig4*−/− mice. At P3 (A–C) and P8 (D–F), cytoplasmic inclusions and vacuolization, leading to extensive cell loss were observed in the spinal cord of *Mtmr2*+/+*Fig4*−/− and *Mtmr2*−/−*Fig4*−/− mutant mice. By counting the number of cells in at least 15 sections of spinal cord per genotype (H, I), a significant decrease in the number of motorneurons (H) and of cells (I) (per mm^2^) was observed at P8 in *Mtmr2*−/−*Fig4*−/− spinal cords as compared to *Mtmr2*+/+*Fig4*−/− (and wild-type). In (H) *Mtmr2*+/+*Fig4*−/− as compared to *Mtmr2*−/−*Fig4*−/−, P = 0.04893; wild-type as compared to *Mtmr2*−/−*Fig4*−/−, P = 0,02107; in (I), *Mtmr2*+/+*Fig4*−/− as compared to *Mtmr2*−/−*Fig4*−/−, P = 0.0134245; wild-type as compared to *Mtmr2*−/−*Fig4*−/−, P = 0.008836. (G) Electron microscopy of a motorneuron showing cytoplasmic electrondense inclusion and vacuoles. Arrows indicate inclusions; arrowheads vacuoles. Bar is 10 µm in (A–F) and 1 µm in (G).

In the cortex and brainstem of *Mtmr2*−/−*Fig4*−/− mice at P3 we noted more cells with vacuoles and inclusions than in *Mtmr2*+/+*Fig4*−/− mice, which were never been observed in wild-type animals (Figure 1D–1I). In particular, in the brainstem of *Mtmr2*−/−*Fig4*−/− mice at P8 the number of neurons carrying pathological abnormalities was significantly increased as compared to *Mtmr2*+/+*Fig4*−/− mice (Figure 1J–1L). We also analyzed the spinal cord of *Mtmr2+/+Fig4−/−* and *Mtmr2−/−Fig4−/−* mice at P3 and P8 (Figure 2A–2F). Vacuolated cells and cells with inclusions were observed, as previously described for the *plt* phenotype, which were not present in wild-type spinal cords [20], [26]. At P8, we observed a significant decrease in the number of motor neurons and cells in *Mtmr2*−/−*Fig4*−/− mice as compared to *Mtmr2*+/+*Fig4*−/− mice (Figure 2H, 2I). These findings demonstrate that loss of Mtmr2 exacerbates the *Mtmr2+/+Fig4−/−* neurodegeneration.

**Figure 3:**
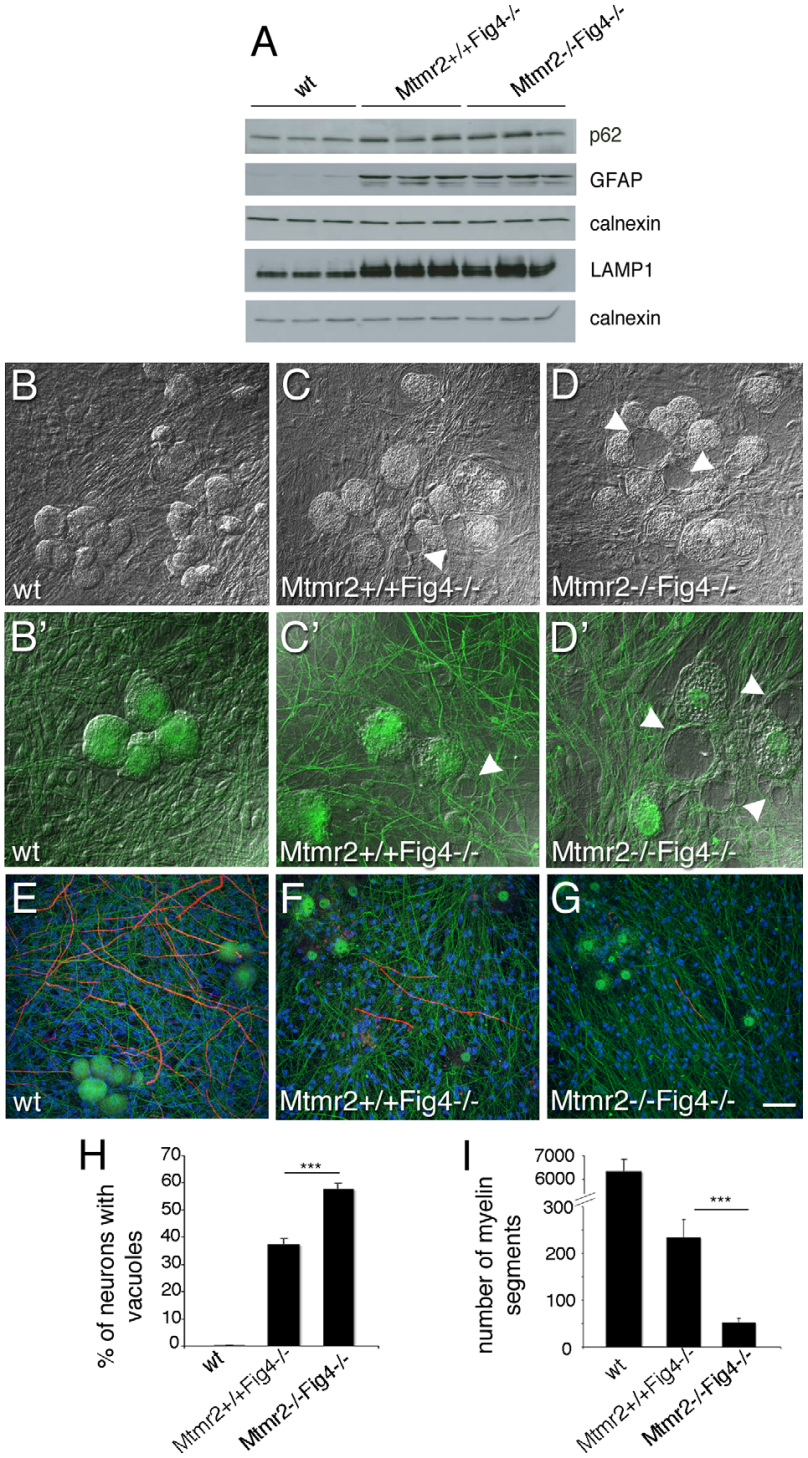
Mtmr2 loss in neurons exacerbates *Fig4*-null neurodegeneration. (A) Western blot analysis of brain extracts from wild-type, *Mtmr2*+/+*Fig4*−/−, and *Mtmr2*−/−*Fig4*−/− mice at P20. P62, GFAP, and LAMP1 are increased in *Mtmr2*+/+*Fig4*−/− as compared to wild-type as already reported for the *plt* mouse. No differences were detected between *Mtmr2*+/+*Fig4*−/− and *Mtmr2*−/−*Fig4*−/−, suggesting that loss of Mtmr2 does not further impair astrocytosis and the block in the autophagic process in astrocytes. (B–G) Dissociated DRG explants from wild-type (B, B′, E), *Mtmr2*+/+*Fig4*−/− (C, C′, F), and *Mtmr2*−/−*Fig4*−/− (D, D′, G) mice, in which mutant Schwann cells were replaced by exogenous wild-type rat Schwann cells. (D, D′) More *Mtmr2*−/−*Fig4*−/− neurons (n = 303 neurons on 523 total neurons) are vacuolated as compared to *Mtmr2*+/+*Fig4*−/− neurons (C, C′; n = 236 on 635 total neurons; P = 7.56271×10^−8^, 16 covers per genotype, and quantification in H). (G) *Mtmr2*−/−*Fig4*−/− co-cultures produced less myelinated MBP-positive segments after 7 days of ascorbic acid treatment (n = 52 segments on 16 coverslips) as compared to *Mtmr2*+/+*Fig4*−/− (F) (n = 234 segments on 12 coverslips; P = 1.9218×10^−5^) and quantification in (I). Both *Mtmr2*+/+*Fig4*−/− and *Mtmr2*−/−*Fig4*−/− explants are severely hypomyelinated as compared to wild-type (E) (n = 6560 segments on 16 coverslips). Green is neurofilament (NF-L); red is myelin basic protein (MBP), staining compact myelin. Arrowheads in (C, D, C′, D′) indicate vacuoles within sensory neurons. Bar in (B–G) is 50 µm.

**Figure 4:**
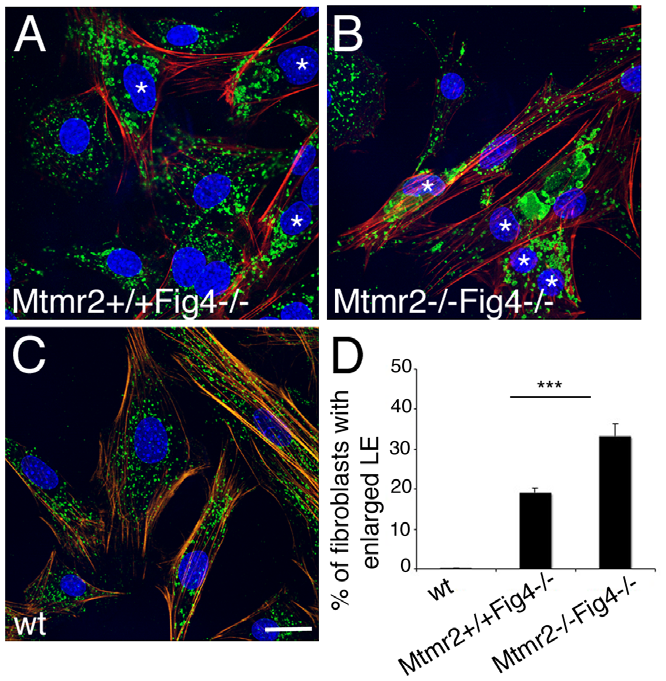
Enlarged late endosomal compartment in primary fibroblasts from *Mtmr2*+/+*Fig4*−/− and *Mtmr2*−/−*Fig4*−/− mutant mice. (A–C) The number of fibroblasts carrying enlarged late endosomal/lysosomal compartment (LAMP1 positive, green) is significantly increased in *Mtmr2*−/−*Fig4*−/− (B) as compared to (A) *Mtmr2*+/+*Fig4*−/−. In red is phalloidin which stains actin filaments. Blue, DAPI. The quantification in (D) was made by counting cells established from three animals per genotype. n = 526 on 17 coverslips for Mtmr2+/+Fig4−/−; n = 583 cells on 20 coverslips for *Mtmr2*−/−*Fig4*−/−; P = 0.000079; confocal microscopy analysis. Asterisks mark cells carrying enlarged late endosomal compartments. Bar in (A–C) is 25 µm.

This effect could be the consequence of loss of Mtmr2 in neurons or in other cells, such as in astrocytes. For example, in the *plt* mouse, a block of autophagy in astrocytes has been reported. In *plt* mice at 1 week of age, the p62 autophagy marker was increased in GFAP-positive astrocytes from brain regions severely affected at later stages, suggesting that autophagy impairment contributes to the pathogenesis [26]. Elevated p62 co-localized with LAMP2-positive late endosomes/lysosomes (LE/LY) in astrocytes, showing that the block of autophagy occurred subsequent to the fusion of autophagosomes with LE/LY [26]. To determine whether loss of Mtmr2 in astrocytes might further impair autophagy, we evaluated p62 levels in total brain extracts from *Mtmr2*+/+*Fig4*−/− as compared with *Mtmr2*−/−*Fig4*−/− mice. Increased p62, LAMP1 and GFAP expression levels were confirmed in *Mtmr2*+/+*Fig4*−/− as compared to wild-type but no differences were detected between *Mtmr2+/+Fig4*−/− and *Mtmr2*−/−*Fig4*−/− double null mice (Figure 3A). This finding indicates that loss of Mtmr2 does not further impair the block in the autophagic process in astrocytes of *Fig4*-null mice.

To further investigate the cell autonomy of the Mtmr2/Fig4 interaction, we established dissociated Schwann cell/DRG neuron co-cultures from *Mtmr2*+/+*Fig4*−/− and *Mtmr2*−/−*Fig4*−/− mice, in which mutant Schwann cells were replaced with exogenous wild-type rat Schwann cells. *Mtmr2*−/−*Fig4*−/− DRG neurons cultured with wild-type Schwann cells were significantly more severely vacuolated (57.8%) as compared to *Mtmr2+/+Fig4−/−* cultures (37.4%) (Figure 3B–3D′ and 3H). This finding provides strong evidence that the loss of Mtmr2 in neurons leads to the worsening of the *Fig4*-null neurodegeneration.

Like neurons, mouse primary fibroblasts (MFs) from *plt* mutants display enlargement and vacuolization of the LAMP2-positive LE/LY compartment [20], [22]. To provide further evidence for functional interaction between MTMR2 and FIG4, we established MF cultures from *Mtmr2+/+Fig4−/−* and *Mtmr2−/−Fig4*−/− mice. By LAMP1 staining and confocal microscopy, we observed that the number of fibroblasts carrying enlarged LE/LY was significantly increased in *Mtmr2−/−Fig4*−/− double mutants as compared to *Mtmr2+/+Fig4−/−* (Figure 4). This finding indicates that Mtmr2 loss exacerbates *Fig4*-null vacuolar phenotype by further impairment of the endo/lysosomal trafficking pathway.

### Mtmr2 exacerbates *Fig4*-null hypomyelination in sciatic nerve

The *plt* mouse phenotype is characterized by a peripheral neuropathy. Loss of large diameter myelinated axons, hypomyelination (reduced myelin thickness), reduced amplitude of compound motor action potential (cMAP) and slowing of the nerve conduction velocity (NCV) have been reported in *plt* mouse nerves at 6 weeks of age [20], [22]. The extent of the NCV reduction in *plt* mice and the presence of demyelinating features in CMT4J patient biopsies such as onion bulbs suggested that FIG4 has also a cell autonomous role in Schwann cells [22].

**Figure 5:**
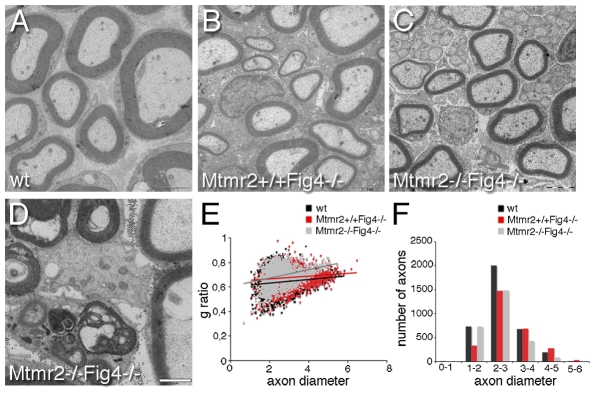
Hypomyelination in *Mtmr2*+/+*Fig4*−/− and *Mtmr2*−/−*Fig4*−/− sciatic nerves. (A–C and E) At P20, the g-ratio (the ratio between axonal diameter and the fiber diameter-axon plus myelin) in *Mtmr2*+/+*Fig4*−/− sciatic nerves (0.674±0.0014) is increased as compared to wild-type (0.6408±0.0013; P = 8.0253×10^−63^), suggesting the presence of hypomyelination, a reduction in myelin thickness. The g-ratio of *Mtmr2*−/−*Fig4*−/− (0.6885±0.0015) sciatic nerves is further increased as compared to *Mtmr2*+/+*Fig4*−/− nerves (P = 2.87033×10^−12^), suggesting that loss of Mtmr2 exacerbates hypomyelination of *Fig4*-null. (D) Electron microscopy of a *Mtmr2*−/−*Fig4*−/− nerve, showing myelin debris and axonal degeneration. (F) The axon diameter distribution is normal in both *Mtmr2*−/−*Fig4*−/− and *Mtmr2*+/+*Fig4*−/−as compared to wild-type, thus excluding loss of axons. In (E, F) axon diameter is in µm scale. From 4 to 5 animals per genotype were analyzed. n = 3450 fibers for wild-type; n = 2650 fibers for *Mtmr2*+/+*Fig4*−/−, and n = 2746 fibers for *Mtmr2*−/−*Fig4*−/−, t-test analysis. Bar is 2 µm in (A–C) and 1 µm in (D).

We investigated sciatic nerves from *Mtmr2*+/+*Fig4*−/− and *Mtmr2*−/−*Fig4−/−* mice. At P3 and P8, mutant sciatic nerves showed a normal development. In both genotypes at P8, Schwann cells often contained cytoplasmic inclusions and occasionally contained vacuoles, which were never observed in wild-type nerves. At P20, the latest time point of survival of *Mtmr2*/*Fig4* double null mice, *Mtmr2+/+Fig4−/−* sciatic nerves were hypomyelinated (thin myelin sheath) with an increased g-ratio (diameter of the axon divided by the diameter of the fiber) as compared to wild-type nerves (Figure 5E). At this stage, sciatic nerves from *Mtmr2−/−Fig4*−/− double null mice were more severely hypomyelinated than *Mtmr2+/+Fig4−/−* mice with a higher g-ratio, demonstrating that Mtmr2 loss exacerbates the neuropathy of *Mtmr2+/+Fig4−/−* mice (Figure 5E). The total number of fibers and the axonal diameter distribution at P20 were not significantly altered in mouse nerves of either genotype (Figure 5F). These observations indicate that the hypomyelination is not a developmental defect related to delayed axonal growth. Hypomyelination may result from a defective axonal/Schwann cell interaction due to the severe neuronal degeneration and/or from the loss of FIG4 in Schwann cells. We thus cultured dissociated DRG neurons from *Mtmr2*−/−*Fig4−/−* and *Mtmr2*+/+*Fig4*−/− mice, seeded with exogenous wild type rat Schwann cells. Following induction of myelination by ascorbic acid treatment, vacuolated DRG neurons from both *Mtmr2*−/−*Fig4*−/− and *Mtmr2*+/+*Fig4*−/− mouse embryos were able to produce myelinated segments, although significantly fewer than wild-type cultures. Moreover, DRG neurons from *Mtmr2*−/−*Fig4*−/− mice cultured with wild-type Schwann cells produced significantly fewer myelinated segments than *Mtmr2*+/+*Fig4*−/− neurons seeded with wild-type Schwann cells (Figure 3E–3G and quantification in panel I). This observation suggests that the hypomyelination of *Mtmr2+/+Fig4−/−* nerves represents at least in part the consequence of impaired Schwann cell-axonal interaction.

### *Fig4* heterozygosity rescues *Mtmr2*-null myelin outfoldings

**Figure 6:**
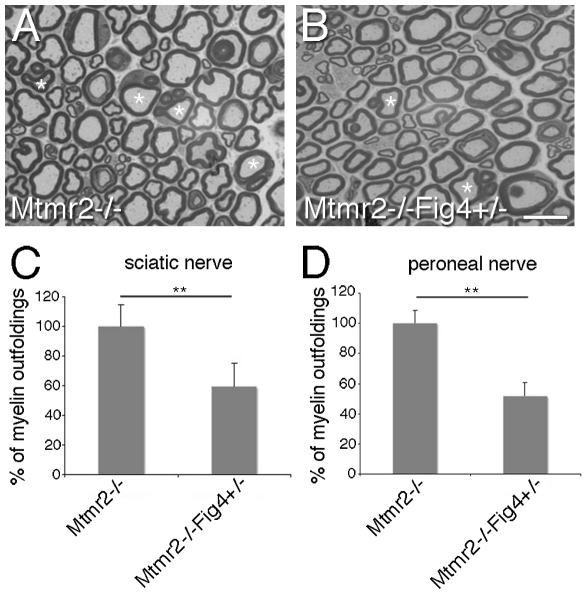
*Fig4* heterozygosity rescues *Mtmr2*-null myelin outfoldings. (A, B) Asterisks indicate myelinated fibers from sciatic nerve carrying myelin outfoldings. (C) Quantification of fibers carrying myelin outfoldings in sciatic nerves from *Mtmr2*−/−*Fig4*+/+ and *Mtmr2*−/−*Fig4*+/− mice and (D) in peroneal nerves, using semithin section analysis. Four animals per genotype were analyzed. The entire nerve section was reconstructed and the number of fibers carrying myelin outfoldings divided for the total number of fibers was counted. P = 0.0097 in (C) and P = 0.0086 in (D). Bar is 10 µm in (A, B).

To further investigate Mtmr2 and Fig4 interaction in the nerve, we evaluated whether loss of Fig4 modifies the myelin outfolding phenotype. Myelin outfoldings in *Mtmr2*-null mice arise around the third to fourth week after birth, and the number of fibers containing myelin outfoldings and loops progressively increases with age (up to 6 months or even later). Since *Mtmr2−/−Fig4*−/−double mutants die before 1 month of age, we compared sciatic and peroneal nerves at 6 months of age from *Mtmr2−/−Fig4+/+* and *Mtmr2−/−Fig4*+/− (*Fig4* heterozygous) mice. Using semithin section analysis, we measured the number of fibers carrying myelin outfoldings in mutant sciatic and peroneal nerves normalized to the total number of fibers. In *Mtmr2−/−Fig4*+/− nerves myelin outfoldings were significantly reduced as compared to *Mtmr2−/−Fig4+/+* mice (Figure 6A–6D). Since loss of Mtmr2 in Schwann cells is both sufficient and necessary to provoke myelin outfoldings, loss of Fig4 in Schwann cells (rather than axons) is likely to account for the rescue of the disease phenotype.

**Figure 7:**
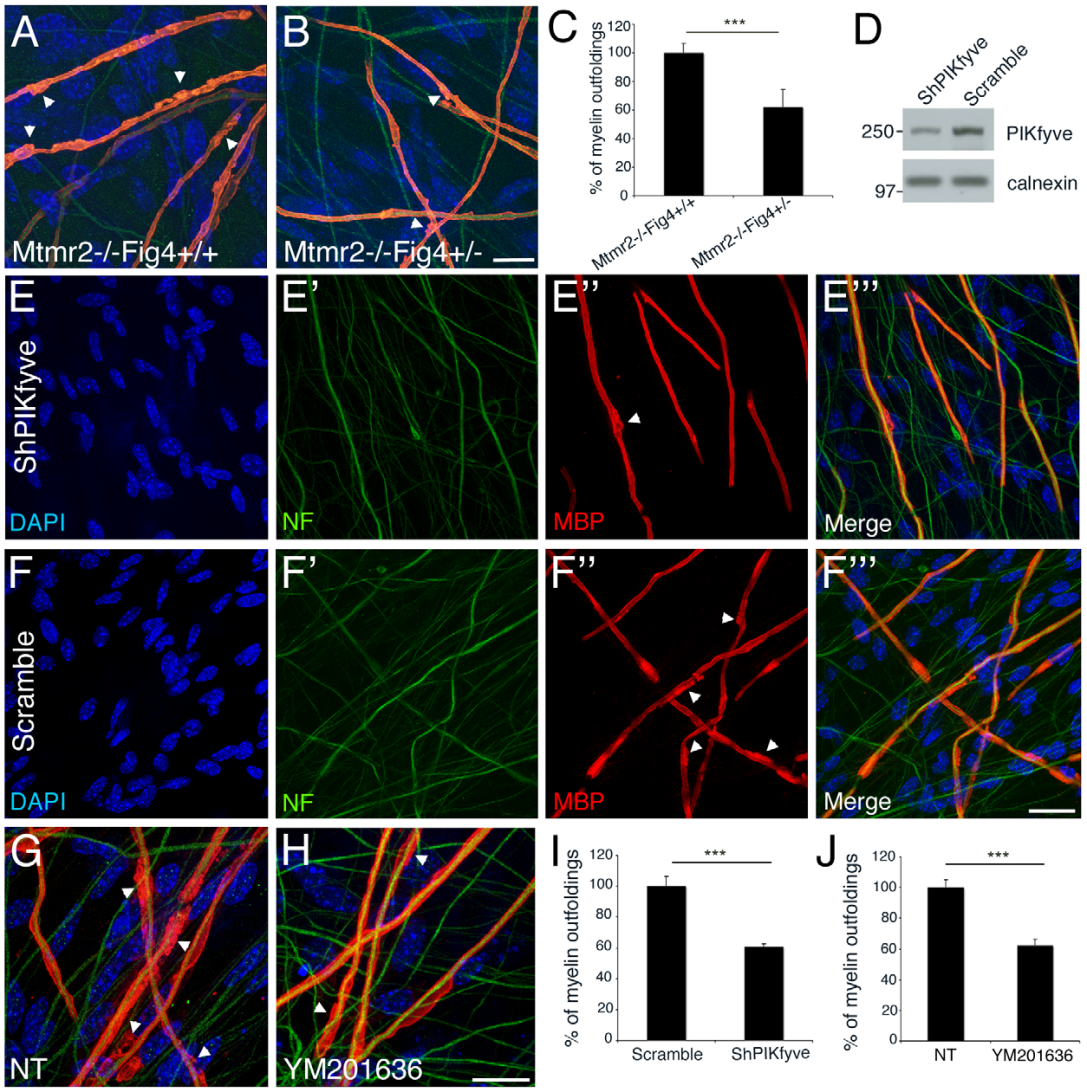
A rebalance of PtdIns(3,5)*P*_2_ rescues *Mtmr2*-null myelin outfoldings *in vitro*. (A, B) Heterozygosity of *Fig4* in *Mtmr2*-null explants rescues myelin outfoldings. (C) The number of MBP positive segments carrying myelin outfoldings on the total of MBP segments on random fields was assessed. *Mtmr2*−/−*Fig4*+/+ n = 383 fibers; *Mtmr2*−/−*Fig4*+/− n = 405 fibers, on at least 5 covers per genotype; P = 1.97554×10^−5^. (D) Decrease of PIKfyve expression on lysates from myelin-forming Schwann cells/DRG explants transduced with PIKfyve shRNA lentiviral vector (LV), as compared to a scramble sequence. (E–F′″ and I) Myelin outfoldings are rescued in *Mtmr2*-null transduced by PIKfyve shRNA LV as compared to cultures transduced with a scramble sequence (PIKfyve, n = 423 and scramble, n = 484; P = 6×10^−6^). (G, H, and J) Myelin outfoldings are rescued in *Mtmr2*-null treated with 70 nM of YM201636 PIKfyve inhibitor as compared to cultures treated with DMSO (YM201636 n = 913; DMSO, n = 857; P = 6.5×10^−7^). Bar is 20 µm in (A, B, E–F″, and G, H). Green is neurofilament (NF-L); red is myelin basic protein (MBP), staining compact myelin. Arrowheads indicate myelin outfoldings.

To further evaluate this finding, we established myelin-forming Schwann cell/DRG neuron co-cultures from *Mtmr2−/−Fig4+/+* and *Mtmr2−/−Fig4*+/− mouse embryos at E13.5 (Figure 7A, 7B). By measuring the number of MBP positive fibers carrying myelin outfoldings in the cultures, we confirmed that *Mtmr2*-null myelin outfoldings were rescued by *Fig4* heterozygosity (Figure 7C).

**Figure 8:**
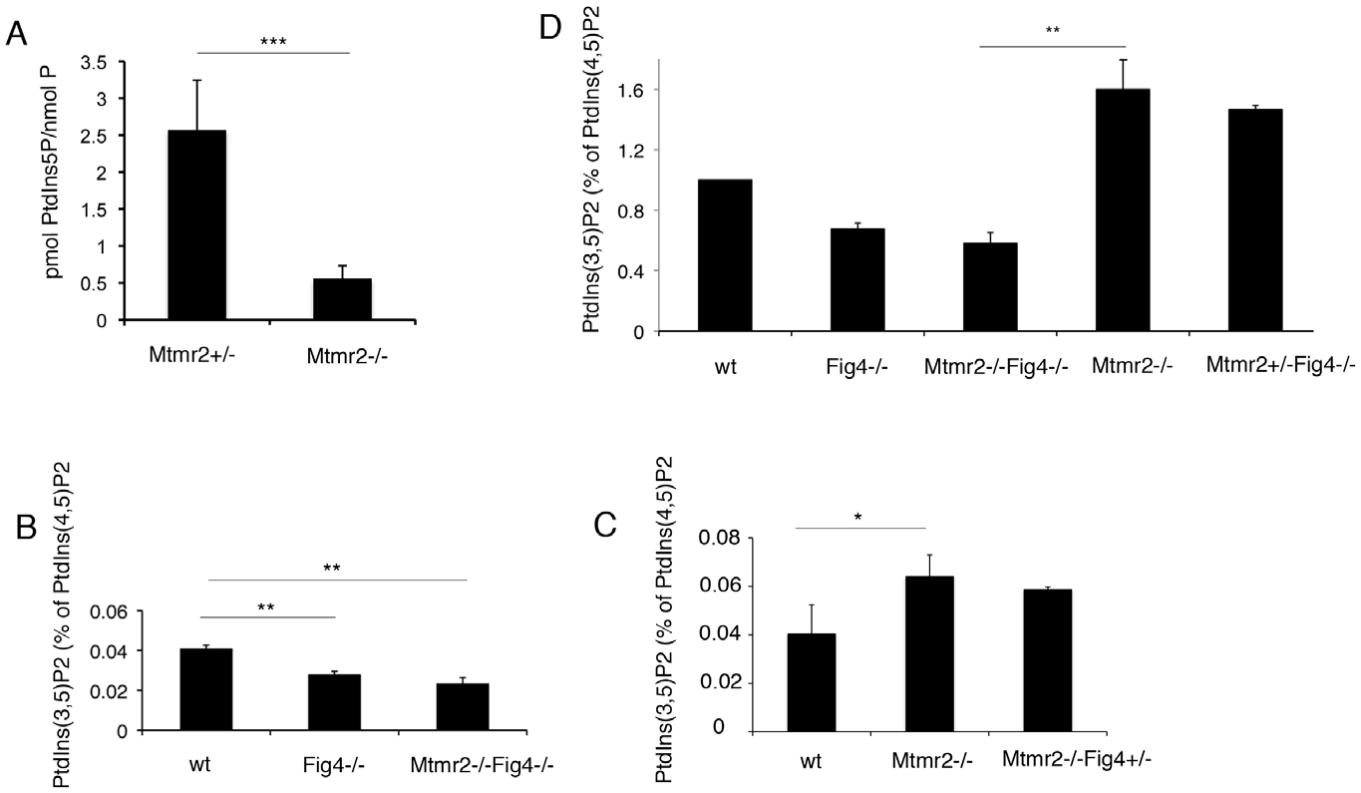
PI measurements from primary fibroblasts and Schwann cell/DRG co-cultures. (A) PtdIns5*P* was measured by *in vitro* mass assay in DRG co-cultures from *Mtmr2*+/− or *Mtmr2*−/− knock-out mice as described in Materials and Methods. Data are expressed as mean ± s.e.m. (n = 3 pools of at least 6 different explants per genotype). *P<0,05. (B–D) Fibroblasts from wild-type, *Mtmr2*+/+*Fig4*−/−, *Mtmr2*−/−*Fig4*+/+, *Mtmr2*−/−*Fig4*−/−, and *Mtmr2*−/−*Fig4*+/− mice were labeled for 16 h with [^32^P]orthophosphate. Lipids were extracted, deacylated and analyzed by HPLC. PtdIns(3,5)*P*_2_ is expressed as percent of PtdIns(4,5)*P*_2_. Data are expressed as mean ± s.e.m. (n = 3). No differences in PtdIns3*P* levels were demonstrated between the different genotypes analyzed (not shown). (B) PtdIns(3,5)*P*_2_ levels were significantly reduced in *Mtmr2*+/+*Fig4*−/− cells as compared to wild-type, thus also confirming previous findings [20], whereas *Mtmr2*+/+*Fig4*−/− and *Mtmr2*−/−*Fig4*−/− cells showed similar levels of this lipid (P = 0.0071 between wild-type and *Fig4*−/−; P = 0.0044, between wild-type and double null cells). (C) Loss of Mtmr2 in *Mtmr2*−/−*Fig4*+/+ cells provoked a significant increase of PtdIns(3,5)*P*_2_ (P = 0.0096), whereas no differences were detected between *Mtmr2*−/−*Fig4*+/+ and *Mtmr2*−/−*Fig4*+/− cells, possibly because heterozygous deletion of *Fig4* leads to small PI changes hardly detectable using this method. (D) Comparison between the two independent experiments showed in (B) and (C), where each value was proportioned to the wild-type belonging to the same experiment and wild-type values were arbitrarily converted to 1 (P = 0.0055 when comparing *Mtmr2*−/−*Fig4*−/− and *Mtmr2*−/−*Fig4*+/+ cells).

Loss of Fig4 in *plt* fibroblasts leads to a significant decrease in PtdIns(3,5)*P*_2_, whereas Mtmr2 loss should lead to an increase in both PtdIns3*P* and PtdIns(3,5)*P*_2_ *in vivo* in the nerve [20]. Indeed, by performing a sensitive *in vitro* mass assay on *Mtmr2*-null Schwann cell/DRG neuron co-cultures, we found that in null cells PtdIns5*P* is significantly reduced (up to 70%) as expected by the loss of MTMR2 3-phosphatase activity on PtdIns(3,5)*P*_2_ (Figure 8A). We hypothesized that the observed rescue by *Fig4* heterozygosity might be the consequence of a restored level of PtdIns(3,5)*P*_2_ in Schwann cells. Heterozygosity of *Fig4* might decrease PIKfyve activity and therefore partially restore PtdIns(3,5)*P*_2_ levels in *Mtmr2*-null cells.

To test this hypothesis, we downregulated either the activity or expression of PIKfyve in *Mtmr2*-null co-cultures to rescue myelin outfoldings. We transduced *Mtmr2*-null co-cultures with lentiviral vectors (LV) carrying PIKfyve shRNA and scored the number of myelinated MBP-positive fibers with myelin outfoldings. Titration of the PIKfyve shRNA LV was previously performed to determine the highest amount of virus which does not significantly affect myelination (Figure S3A, S3B). We found that myelin outfoldings were significantly rescued by downregulating PIKfyve expression (Figure 7D–7F′″ and 7I). We also treated *Mtmr2*-null cultures with a specific pharmacological inhibitor of PIKfyve, YM201636 [27]–[29]. Titration of the compound was performed to determine the maximum amount of YM201636 that does not inhibit myelination (Figure S3A, S3B). Seventy nM final concentration of YM201636 was freshly added to the culture media every other day together with ascorbic acid to achieve full myelination. A significant reduction of myelin outfoldings was confirmed in *Mtmr2*-null cultures treated with YM201636 as compared with DMSO alone (Figure 7G, 7H, 7J). The data suggest that reduction of the level of PtdIns(3,5)*P*_2_, either by heterozygosity for *Fig4* or by inhibition of PIKfyve, corrects the myelin abnormality of *Mtmr2*-null cells This result predicts that the level of PtdIns(3,5)*P*_2_ may be elevated in *Mtmr2*-null cells.

### Phospholipid analysis in mouse fibroblasts

To correlate MTMR2 and FIG4 functional interaction with changes in PI levels, we measured PtdIns3*P* and PtdIns(3,5)*P*_2_ levels from wild-type; *Mtmr2*−/−*Fig4*+/+; *Mtmr2*+/+*Fig4*−/−; *Mtmr2*−/−*Fig4*−/−, and *Mtmr2*−/−*Fig4*+/− fibroblasts by metabolic labeling and HPLC analysis (Figure 8). PtdIns3*P* levels were similar in all the genotypes analyzed (data not shown). In mammalian cells, PtdIns3*P* generation and turnover are controlled by multiple redundant pathways, so that ablation of one particular enzyme such as myotubularins does not necessarily result in an imbalance of PtdIns3*P*, as already reported [9], [30], [31].

On the other hand, we found that loss of Fig4 in *Fig4*-null fibroblasts results in a significant decrease of PtdIns(3,5)*P*_2_ as compared to control cells, thus confirming previous findings [20] (Figure 8B). As also suggested by the *in vitro* mass assay performed on *Mtmr2*-null myelin-forming co-cultures (Figure 8A), loss of Mtmr2 in *Mtmr2*-null fibroblasts leads to a significant increase in PtdIns(3,5)*P*_2_ level, consistently with the 3-phosphatase activity of MTMR2 (Figure 8C). Moreover, PtdIns(3,5)*P*_2_ was equally reduced in *Fig4*−/− and in *Mtmr2*−/−*Fig4*−/− cells (Figure 8B), possibly because the PtdIns(3,5)*P*_2_ substrate is already severely affected by loss of Fig4, and Mtmr2 acts downstream of Fig4 in the control of this lipid level.

To support the hypothesis that myelin outfoldings in *Mtmr2*−/−*Fig4*+/− co-cultures were rescued because of restored PtdIns(3,5)*P*_2_ levels, we also measured PtdIns(3,5)*P*_2_ in *Mtmr2*−/−*Fig4*+/+ and *Mtmr2*−/−*Fig4*+/− fibroblasts. However, PtdIns(3,5)*P*_2_ did not differ in *Mtmr2*−/−*Fig4*+/+ and *Mtmr2*−/−*Fig4*+/− fibroblasts (Figure 8C). Small changes in PtdIns(3,5)*P*_2_ levels due to loss of 50% of phosphatase expression may be below the level of detection of this method.

Overall, these findings indicate that Mtmr2 and Fig4 control PtdIns(3,5)*P*_2_ with opposite effects. If Fig4 is totally absent and PtdIns(3,5)*P*_2_ is low, the absence of Mtmr2 which dephosphorylates PtdIns(3,5)*P*_2_ has no influence. On the other hand, when PtdIns(3,5)*P*_2_ is high due to loss of Mtmr2, a partial reduction in PIKfyve activity due to heterozygosity of *Fig4* might lead to PtdIns(3,5)*P*_2_ rebalance and rescue of myelin outfoldings.

Finally, we tested for interaction between phosphatases using a pull-down assay. GST-MTMR2 was not able to pull-down Fig4 from brain or isolated rat Schwann cell lysates, suggesting that the functional interaction between MTMR2 and FIG4 demonstrated here is not mediated by physical interaction between the two proteins (Figure S3C–S3E).

### Mammalian MTMR2 converts PtdIns(3,5)*P*_2_ and PtdIns3*P* in yeast

**Figure 9:**
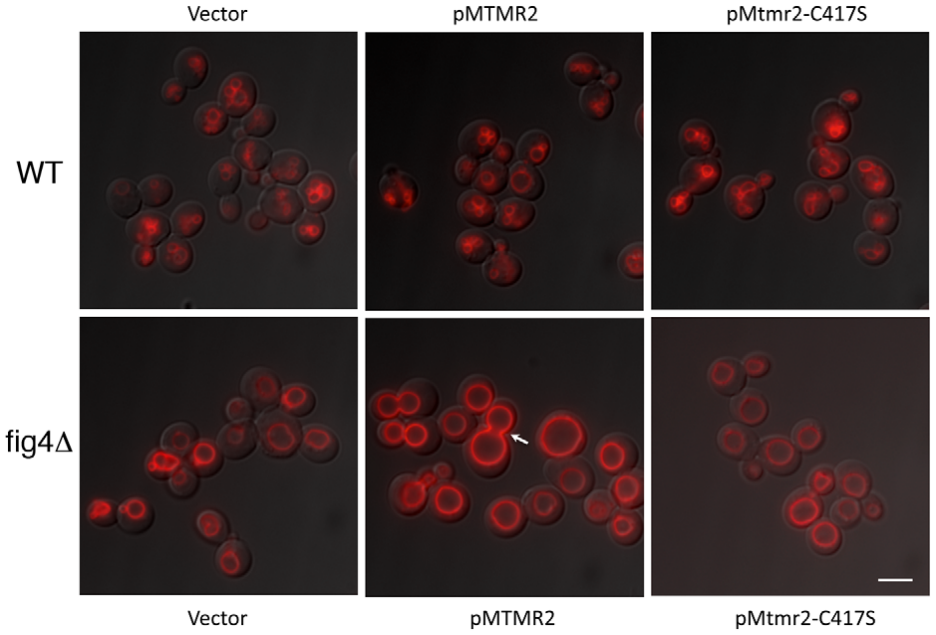
MTMR2 and FIG4 interaction in yeast. Expression of human FLAG-MTMR2 in the mutant yeast strain *fig4*Δ, caused enlarged vacuoles and a defect in vacuole fission. Wild-type and *fig4*Δ expressing pVT102U (vector), pVT102U-*MTMR2* or pVT102U-*MTMR2-C417S*, were grown to mid-log phase and the vacuole membrane was labeled with SynaptoRed C2. Arrow indicates an example of a bud vacuole that has not properly separated from the mother vacuole. Scale bar, 6 µm.

**Figure 10:**
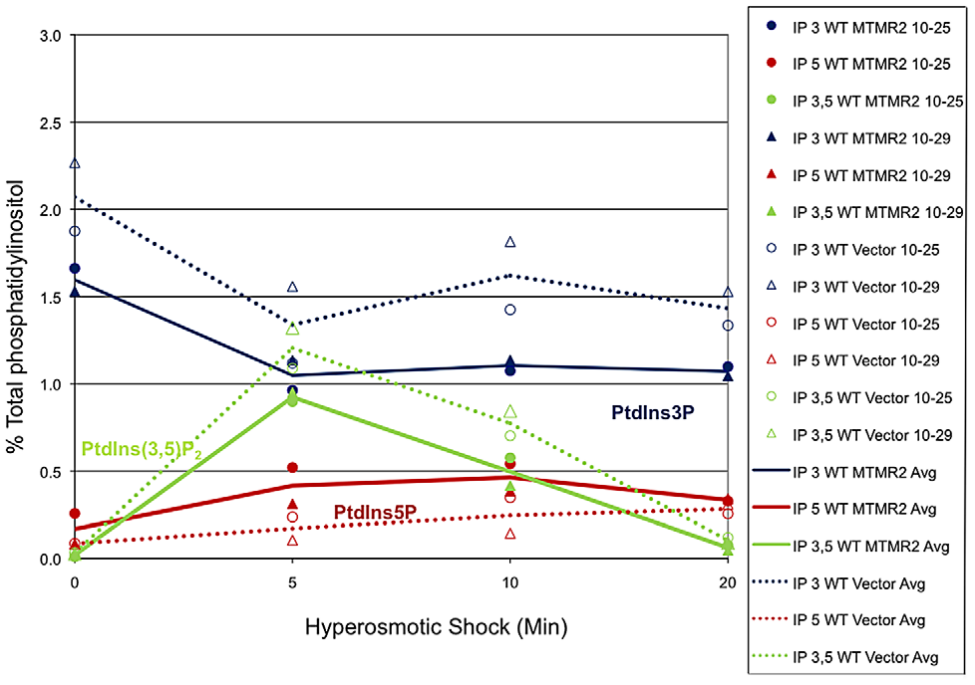
MTMR2 3-phosphatase activity in yeast. Mouse MTMR2 has 3- phosphatase activity *in vivo* and converts PtdIns(3,5)*P*_2_ and PtdIns3*P* to PtdIns5*P* and phosphatidylinositol, respectively. Expression of MTMR2 in yeast causes a decrease in PtdIns3*P* and PtdIns(3,5)*P*_2_ as well as an increase in PtdIns5*P*. Mouse MTMR2 cDNA was subcloned into a yeast expression vector, pVT102U (vector) to express MTMR2 from the ADH promoter, pVT102U-*MTMR2*. Yeast expressing MTMR2 or with vector alone were labeled with ^3^H-inositol for 18 h. Cells were exposed to 0.9 M NaCl for the times indicated, Lipids were extracted, deacylated, and the corresponding glycerol-inositol phosphates were quantitated by high-pressure liquid chromatography, reported as the percent of total [3H]phosphatidylinositol extracted. Two independent experiments are shown.

The mutant yeast strain *fig4*Δ displays enlarged vacuoles caused by reduced PtdIns(3,5)*P*_2_, which in yeast controls the homeostasis of the vacuole (the lysosomal compartment). To further test Mtmr2 function, and further test functional interactions between Mtmr2 and Fig4, we transformed FLAG-MTMR2 in the mutant yeast strain *fig4*Δ. Overexpression of wild-type MTMR2 in *fig4*Δ caused a further enlargement of the vacuolar compartment and defects in vacuole fission whereas the catalytically inactive mutant FLAG-MTMR2C417S did not cause these changes (Figure 9). To determine the substrates and products of mammalian MTMR2 in yeast, we measured phosphorylated phosphoinositide lipid levels from cells expressing FLAG-MTMR2 as compared to the vector alone. To enhance the sensitivity of the assay, we subjected the yeast to hyperosmotic shock. In wild-type yeast, this results in a transient increase in PtdIns(3,5)*P*_2_ levels (green line) and concomitant decrease in PtdIns3*P* (blue line) (Figure 10, 5 min time point). If MTMR2 acts on PtdIns(3,5)*P*_2_, then there should be a decrease in PtdIns(3,5)*P*_2_ and a corresponding increase in PtdIns5*P*. Moreover, if MTMR2 acts on PtdIns3*P* there will be a decrease in that lipid as well. Each of these changes was observed (Figure 10, solid lines and Table S1). These findings demonstrate that MTMR2 acts on both PtdIns(3,5)*P*_2_ and PtdIns3*P* in yeast, and strongly suggest that MTMR2 acts on both of these substrates in mammalian cells as well. These observations support the hypothesis that MTMR2 and FIG4 coordinately regulate the PtdIns3*P*-PtdIns(3,5)*P*_2_ pathway *in vivo*.

## Discussion

The MTMR2 3-phosphatase activity toward PtdIns3*P* and PtdIns(3,5)*P*_2_ has been demonstrated by a number of studies using recombinant MTMR2 *in vitro* as well as conventional cell lines overexpressing MTMR2 [10]–[16]. Overexpressed MTMR2 has been co-localized with Rab7 in A431 cells at the level of late endosome/lysosomes, where PtdIns(3,5)*P*_2_ is generated [16]. Interestingly, another phospholipid phosphatase, FIG4/SAC3, is involved in both the dephosphorylation and the production of PtdIns(3,5)*P*_2_ and is mutated in autosomal recessive demyelinating CMT4J neuropathy [20]. Loss of Fig4 in mouse provokes the *plt* phenotype characterized by massive neurodegeneration and peripheral neuropathy. In *Fig4*-null fibroblasts a decrease in PtdIns(3,5)*P*_2_ has been demonstrated, suggesting that Fig4 promotes PtdIns(3,5)*P*_2_ production by PIKfyve activation or stabilization [20]. Thus, MTMR2 and FIG4 could have opposite effects in the control of PtdIns(3,5)*P*_2_.

To explore the biological role of MTMR2 phosphatase activity in the nerve *in vivo*, we generated a *Mtmr2*/*Fig4* double null mutant. Analysis of these mice provides evidence that Mtmr2 and Fig4 functionally interact in neurons, fibroblasts, and Schwann cells. Loss of Mtmr2 reduces the viability and exacerbates the neurodegeneration of *Fig4*-null mice.

These results also provide the first evidence for a role for MTMR2 in neurons *in vivo*, consistent with the marked axonal loss in CMT4B1 patients [32]. Although conditional ablation of Mtmr2 in motorneurons in mice did not reveal signs of axonal degeneration or neuronopathy, a cell autonomous role of Mtmr2 in neurons was not excluded since axonopathies are length dependent and not easily reproduced in mice [18]. Interestingly, a role for MTMR2 in neurons *in vitro* has been recently reported suggesting that Mtmr2 is localized to excitatory synapses of central neurons via direct interaction with the PSD-95 scaffolding protein [33]. Knockdown of Mtmr2 in cultured neurons markedly reduced excitatory synapse density and function and it was proposed that the MTMR2/PSD95 complex contributes to the maintenance of excitatory synapses by inhibiting excessive endosome formation and destructive endosomal traffic to lysosomes.

Here, we assessed MTMR2 and FIG4 interaction in yeast and found that overexpression of MTMR2 reduces both PtdIns3*P* and PtdIns(3,5)*P*_2_ leading to an increase in vacuole size in the *fig4*Δ mutant. These findings support the *in vivo* role of MTMR2 as a 3-phosphatase that acts on both PtdIns3*P* and PtdIns(3,5)*P*_2_.

**Figure 11:**
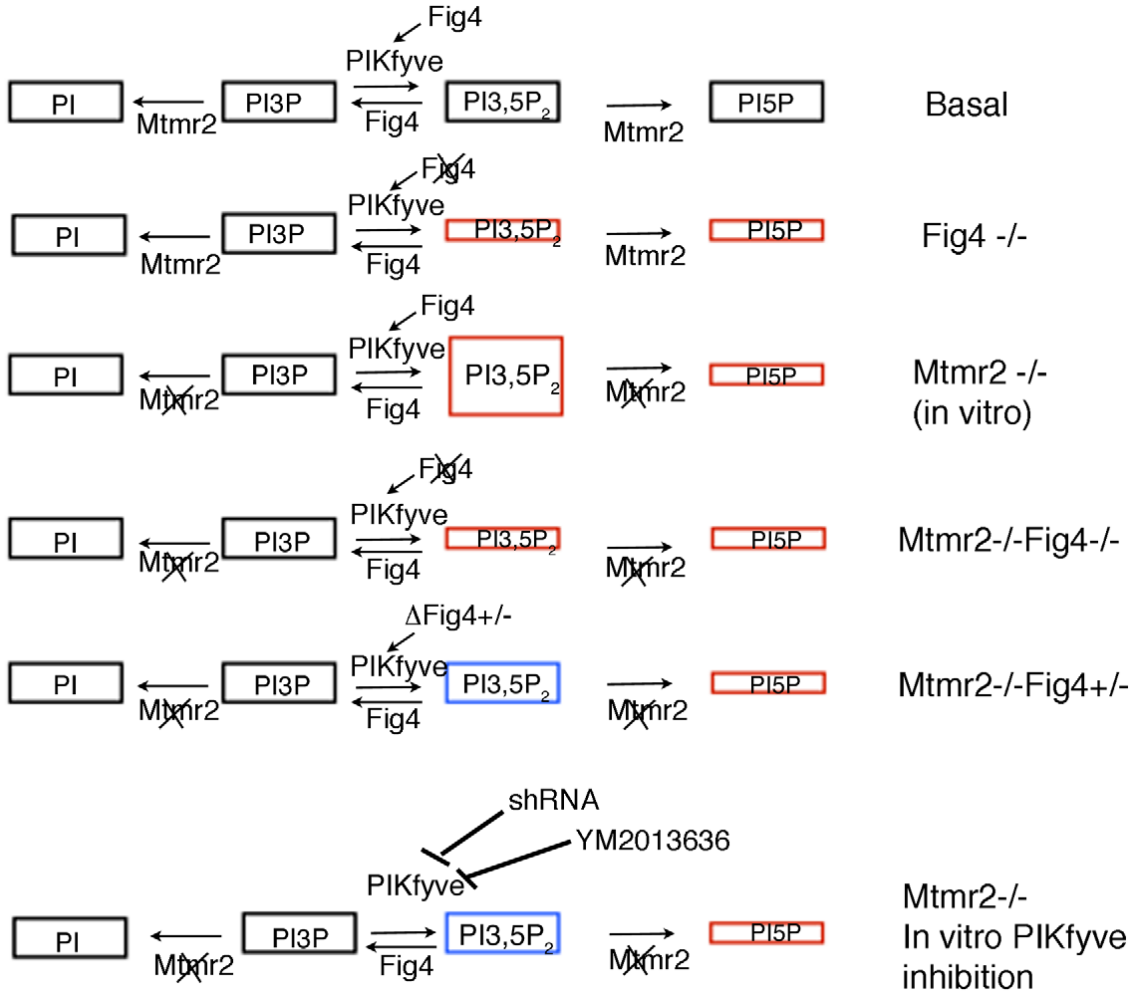
Model of MTMR2 and FIG4 phosphatase activities in the control of PtdIns3*P*- PtdIns(3,5)*P*_2_ metabolism. In red, altered levels of PIs due to loss of MTMR2 and/or FIG4 are depicted. In blue, rebalanced levels of PtdIns(3,5)*P*_2_ in *Mtmr2*-null cells upon *Fig4* heterozygosity and downregulation of either PIKfyve expression or inhibition of its activity are shown.

*Fig4* heterozygosity rescues myelin outfoldings due to Mtmr2 deficiency both *in vivo* and *in vitro*, thus providing evidence of the Fig4 and Mtmr2 interaction in Schwann cells as well as neurons. Loss of Mtmr2 specifically in Schwann cells provokes myelin outfoldings. The presence of cytoplasmic inclusions in Schwann cells and the reduced NCV in the *Fig4*-null mouse, and the typical demyelinating features (onion bulbs) of CMT4J patients, all strongly support a Schwann cell autonomous role for Fig4. But how does loss of Fig4 in Schwann cells rescue *Mtmr2*-null myelin outfoldings? We hypothesized that a 50% reduction of Fig4 might be sufficient to rebalance the PtdIns(3,5)*P*_2_ elevation in *Mtmr2*-null cells (Figure 11), thus reducing myelin outfoldings. MTMR2 loss should lead to an increase of both PtdIns3*P* and PtdIns(3,5)*P*_2_, whereas FIG4 loss reduces PtdIns(3,5)*P*_2_ levels. In agreement with this model, we observed that downregulation of PIKfyve expression or inhibition of its activity in *Mtmr2*-null co-cultures reduced myelin outfoldings, as also observed with *Fig4* heterozygosity (Figure 11). Our results therefore suggest that imbalance of PtdIns(3,5)*P*_2_ is at the basis of altered longitudinal myelin growth and formation of myelin outfoldings. The observed rescue of myelin outfoldings is likely mediated by restored PtdIns(3,5)*P*_2_ rather than PtdIns5*P*. PtdIns5*P* may be produced *via* dephosphorylation of PtdIns(3,5)*P*_2_ by MTMRs, and can also be generated, at least *in vitro*, by PIKfyve acting on phosphatidylinositol [34]. Therefore, *Fig4* heterozygosity in *Mtmr2*-null cells would lead to a further reduction in PtdIns5*P* rather than restoration, as for PtdIns(3,5)*P*_2_.

PtdIns(3,5)*P*_2_ is thought to be localized to EE and the limiting membranes of LE/LY, although it cannot be excluded that this lipid might also be generated at other membranes. The lack of specific probes to detect PtdIns(3,5)*P*_2_ prevents the definition of other membrane localization [35], [36]. Our studies raise the question of how dys-regulation of PtdIns(3,5)*P*_2_ leads to aberrant longitudinal myelin growth. Excessive longitudinal myelin growth and myelin outfoldings might arise as a consequence of reduced endocytosis/recycling and degradation or as a consequence of increased exocytosis. One can speculate that increased PtdIns(3,5)*P*_2_ due to loss of MTMR2 might favor exocytosis from the LE/LY compartment during myelin biogenesis. However, this mechanism, which has been recently suggested to occur in oligodendrocytes [37], accounts for the assembly of myelin components during the active phase of myelination. In myelin outfoldings, myelin thickness is normal, so a more subtle mechanism of regulation would be involved. Increased PtdIns(3,5)*P*_2_ might alter autophagy dynamics. However, we did not observe any change on LC3II/I ratio and/or p62 levels in *Mtmr2*-null nerves or in myelin-forming DRG co-cultures (unpublished results).

Alternatively, MTMR2 may favor endocytosis and counteract exocytosis during later stages of myelin biogenesis. The myelin outfoldings may thus arise as a consequence of the loss of negative control on the amount of membrane produced during myelination. Another alternative is that MTMR2 might control endocytosis of specific transmembrane proteins linking Schwann cell plasma membrane to the axonal plasma membrane, which then act as signaling molecules to control longitudinal myelin growth. Note that myelin outfoldings often contain axoplasm and axons branches at paranodal regions thus following myelin membrane outgrowth [17]. Along these lines, enhanced surface localization of putative adhesion molecules due to loss of Mtmr2-mediated endocytosis might result in the loss of control of myelin elongation and thus in myelin outfoldings. Other members of the MTMR family seem to possess similar biological functions. MTMR4 was recently demonstrated to regulate the sorting of endosomal cargos into recycling endosomes [38]. In *C. elegans*, MTM6 and MTM9 were found to be involved in endocytosis [39] whereas *Drosophila* Mtm (homologous to catalytically active MTM1, MTMR1, and MTMR2) regulates both actin-based plasma membrane morphogenesis and the endosomal influx toward the endo-lysosomal axis [40]. Whether and how MTMR2 might regulate endocytosis in Schwann cells during postnatal development remains to be assessed.

## Materials and Methods

### Ethics statement

All experiments involving animals were performed in accordance with Italian national regulations and covered by experimental protocols reviewed by local Institutional Animal Care and Use Committees.

### Mice

*Mtmr2*-null mice were backcrossed for at least 5 generations to strain C57BL/6N.

*Fig4*+/− heterozygous mice were maintained on the recombinant inbred line CB.plt derived predominantly from strains CAST/Ei and C57BL/6J (25%) [25].

Heterozygous *Fig4*+/− males were crossed with *Mtmr2*-null females to obtain *Mtmr2*+/−*Fig4*+/− double heterozygous mice. Double heterozygotes were crossed to generate *Mtmr2*−/−*Fig4*−/− double null mice as well as *Mtmr2*−/−*Fig4*+/− mice for analysis. Genotyping was performed as described [17], [20].

### Morphological analysis

Semithin morphological analysis was performed as described previously [41]. For morphometric analysis in brainstem at P8, neuronal damage was evaluated in the facial nucleus at the level of the upper medulla oblongata (Bregma −5.88). For each experimental sample, microscopic images (130 um×90 um, nine images per slide, three slides for each brain) were taken with a digital camera and processed by Adobe Photoshop 7.0 software. To be counted, a cell (diameter >20 µm) had to be located in the facial nucleus and 100–150 cells were scored per section. Cells with abnormal cytoplasm vacuolization were scored as pathological. The average percentage of normal and damaged neurons for each sample was considered for each experimental group to represent the neuronal density. Counts were performed in double blind by 2 investigators on slides with a number-code system, and results were analyzed.

The number of motorneurons and of total cells in spinal cord was assessed by performing at least 15 sections for each spinal cord from three animals per genotype as before and by counting the number of cells per area-cell density (mm^2^).

The proportion of fibers carrying myelin outfoldings in *Mtmr2*-null nerves as compared to *Mtmr2*-null mice with *Fig4*+/− heterozygosity was determined by measuring the number of fibers carrying myelin outfoldings normalized to the total number of axons per section (the entire nerve section was reconstructed).

Ultrathin morphological analysis was conducted as reported previously [41]. For morphological analysis, three to five animals were evaluated at each time point in most cases.

### Primary mouse fibroblast (MF) culture

MFs were established at P3 from tails and legs chopped in pieces and incubated after PBS washing with RPMI medium and 1 mL Collagenase Type II (Stock = 2000 U/mL in 1×PBS, Worthington, LS004204) overnight at 37°C. The next day, cells were plated in RPMI-1640 with 15% FBS/1× L-Glutamine/1× Pen/Strep. Cells were subjected to only two-three passages to allow maximum efficiency of metabolic labelling for PI measurement.

### Phospholipid analysis

Fibroblasts were labeled for 16 h in phosphate free DMEM (Invitrogen) containing 200 µCi/ml [^32^P]orthophosphate (Perkin Elmer). Lipids were extracted, separated on Silica gel G60 plates and analyzed by HPLC as described previously [42].

PtdIns5*P* was quantified by mass assay as described [43]. Briefly total lipids were extracted from duplicate or triplicate plates of DRG co-cultures from *Mtmr2*+/− or *Mtmr2*−/− knock-out mice and separated on Silica gel G60 plate. Monophosphorylated PIs were scraped, eluted from silica and assessed for PtdIns(4,5)*P*_2_ formation *in vitro* using the recombinant specific PIP4KIIalpha and [gamma-^32^P] ATP.

### Antibodies

For western blot analysis and immunohistochemistry the following antibodies were used: rat anti-LAMP1 (Iowa Hybridoma bank), Guinea pig anti-P62 (Progen), rabbit anti-GFAP (Sigma), rabbit anti-MAG (kindly provided by Dr. J. Salzer), rat anti-MBP (kindly provided by Dr. V. Lee), mouse anti-MBP (Covance), rabbit anti-NF-L (Chemicon), mouse anti-tubulin (Sigma), and mouse anti-FIG4 (NeuroMab).

### Schwann cell/DRG neuron co-cultures

Myelin-forming Schwann cell/DRG neuron co-cultures were established from E13.5 mouse embryos as previously described [19], [44]. Myelination was induced by treatment for 15 days with ascorbic acid (final concentration, 50 µg/ml) (Sigma-Aldrich). Dissociated Schwann cell/DRG neuron co-cultures were established as described but DRGs were first incubated with trypsin (0.25%) for 45 min at 37°C. Cells were also mechanically dissociated and then plated at a concentration of one to two DRGs per glass coverslip. Isolated rat Schwann cells were prepared as reported previously and cultured using DMEM with 10% of fetal calf serum, 2 ng/ml recombinant human neuregulin1-b1 (R&D Systems), and 2 mM forskolin (Calbiochem).

YM201636 was provided by Symansis. A titration of the compound starting from 800 nM until 30 nM was performed on co-cultures to select the maximum amount of coumpound which did not affect myelination. As already described [27], [28], 400 or 800 nM of compound provoked extensive cell vacuolization after overnight incubation. YM201636 was provided to co-cultures at 70 nM every other day together with ascorbic acid for 13 days to achieve full myelination.

### Immunohistochemistry

Schwann cell/DRG neuron co-cultures were fixed for 15 min in 4% paraformaldehyde, permeabilized for 5 min in ice-cold methanol at −20°C, blocked for 20 min with 10% normal goat serum (Dako), 1% bovine serum albumin (BSA) (Sigma-Aldrich), and then incubated with primary antibody for 1 h. After extensive washing, the coverslips where incubated with the secondary antibody for 30 min, washed, and mounted. For double immunostaining with anti-NF-L and anti- MBP antibody, the coverslips were blocked with 1% BSA, 10% NGS for 20 min on ice, and primary antibodies were incubated overnight at 4°C.

For LAMP1 staining, fibroblasts were permeabilized using 0.1% saponin after fixation. For immunolabeling, secondary antibodies included fluorescein-conjugated (FITC) and rhodamine (tetramethylrhodamine isothiocyanate) (Jackson ImmunoResearch). Coverslips were analyzed using TCS SP5 laser-scanning confocal (Leica) or Olympus BX (Olympus Optical) fluorescent microscope, and Zeiss Axiovert S100 TV2 with Hamamatsu OrcaII-ER.

### Analysis of myelination

To quantify the amount of myelination, the number of MBP positive segments in each explant/coverslip was assessed. As myelination is also a function of the amount of neurites/axons and of the Schwann cell number in the culture, the network of NF-L positive filaments and the number of Schwann cells (DAPI) were also evaluated in each explant. To quantify MBP-positive fibers displaying myelin outfoldings, at least 200 MBP-positive myelinated fibers per explant/coverslip were evaluated, in at least ten different explants/coverslip. The percentage of MBP-positive fibers showing myelin outfoldings among the total number of MBP-positive fibers was counted.

### Analysis of fibroblasts with enlarged late endosome/lysosomes

Fibroblasts were stained using LAMP1 antibody and images were acquired using a confocal microscope. Images were then processed using the Image J software and those cells displaying almost all LAMP1 positive endosomes bigger than 1.67 µm (only occasionally observed in wild-type cells) were considered as carrying enlarged late endosome/lysosomes.

### Imaging and statistical analysis

Micrographs were acquired using a digital camera (Leica F300), and figures were prepared using Adobe Photoshop, version 7.0 and 8.0 (Adobe Systems). Statistical analysis was performed using the Student *t* test; two tails, unequal variants, and alpha = 0.005 were used. Error bars in the graphs represent SEM.

### Lentiviral vector (LV) preparation

To downregulate PIKfyve expression, a shRNA cloned into pLKO.1 LV (human U6 promoter) without a GFP reporter was used (clone ID TRCN0000150081). Non-concentrated LVs were used for RNA interference. The transfer constructs were transfected into 293FT cells together with packaging plasmids Δ8.9 and pCMV-VSGV using Lipofectamine 2000 (Invitrogen). As control, a vector encoding a shRNA to a nonspecific sequence (luciferase) was used. Viral supernatants were collected 48 h after transfection, centrifuged at 3000 rpm for 15 min, and frozen at −80°C.

To check for PIKfyve depletion, freshly plated rat Schwann cells (10^6^ cells per 100-mm plate) were incubated with the LVs in DMEM, 10% FBS, and 2 mM L-glutamine plus forskolin and rhNRG-1 (EGF domain, R&D). Cells were expanded for an additional week and maintained in MEM, 10% FBS, 2 mM L-glutamine and 2 µM forskolin before use. A western blot using a anti-PIKfyve antibody (Santa Cruz) was performed. Using non-concentrated LV, transduction of Schwann cell/DRG neuron co-cultures was performed 4–5 days after dissection by incubating the cells with LVs overnight. Cells were then supplemented with C-media, and myelination was induced after 2 days.

### Glutathione S-transferase–binding assays

Glutathione *S*-transferase (GST) fusion proteins were expressed in *Escherichia coli* BL21 cells and purified directly from bacterial extract on glutathione-Sepharose 4 Fast Flow beads. Rat isolated Schwann cells and mouse brains were homogenated, and protein lysates were prepared using a binding buffer with 1%NP-40, 50 mM Tris buffer, pH 7.4, 10% glycerol, 100 mM NaCl, 10 mM NaF, 1 mM Na-vanadate. Equal amounts of protein lysates were incubated for 4 h at 4°C with immobilized GST fusion proteins and GST as control. After three washes with a buffer containing 0.5% NP-40, the pellets were dissolved in SDS sample buffer and analyzed by SDSPAGE and immunoblotting. To show the relative amount of GST fusion proteins used, beads were dissolved in SDS sample buffer and analyzed by SDS-PAGE, and the gel was stained with Coomassie.

### Yeast analysis and phospholipid measurement in yeast

Yeast cells were labeled with SynaptoRed C2 (Biotium, Inc., CA). 0.1 units of cells (at 600 nm) were collected and resuspended in 250 µl fresh media. 6 µl of SynaptoRed C2 (10 µg/µl dissolved in dimethyl sulfoxide) was added to the cells and incubated at 24°C for 1 hour. Cells were then washed 2 times with fresh media and chased for 2.5 hours. Fluorescence and differential interference contrast (DIC) images were generated using a DeltaVision RT Microscope System (Applied Precision, WA). Images were processed using Softworx and Adobe Photoshop.

Measurement of phosphoinositide levels were performed as described previously [45]. Cells were grown in selective media to mid-log phase, harvested, washed, and resuspended in synthetic media lacking inositol. 1–4×10^6^ cells were inoculated into 5 ml of media lacking inositol containing 5 µCi of myo-[2-^3^H]-inositol. Cells were labeled for 18 h at 24°C, harvested by centrifugation, washed, and resuspended in 100 µl of inositol-free media. For hyperosmotic shock, an equal volume of 1.8 M NaCl was added to cells (for a final concentration of 0.9 M NaCl) and the resulting suspension was incubated at 24°C for the times indicated. 800 µl of ice cold 4.5% perchloric acid [46] was added to the cells. Cells were lysed in the presence of 0.5-mm zirconia beads (Biospec, Bartlesville, OK) on a Beadbeater (Biospec) for three cycles of 2 min at room temperature followed by 2 min on ice. Cell extracts were centrifuged at 14,000 rpm for 10 min at 4°C. Precipitates were washed with 1 ml of 100 mM EDTA, centrifuged 14,000 rpm for 10 min at 4°C, and resuspended in 50 µl of sterile distilled deionized water.

Lipids were deacylated by treatment with methylamine [47].1 ml methylamine reagent (10.7% methylamine, 45.7% methanol, 11.4% n-butanol) was added to each sample and incubated at 55°C for 1 h. Samples were dried in a SpeedVac and the pellets were resuspended in 300 µl of sterile water, centrifuged at 14,000 rpm for 2 min and the supernatants were transferred to new Eppendorf tubes. 300 µl of butanol/ethyl ether/formic acid ethyl ester (20∶4∶1) was added to each. The samples were vortexed and centrifuged at 14,000 rpm for 2 min. The aqueous phase (bottom layer) was transferred to new tubes and the extraction was repeated. At the end of the second extraction the aqueous phase was dried in a SpeedVac. Samples were resuspended in 20 µl of sterile water and 15 µl of each was analyzed by HPLC using an anion exchange, PartisphereSAX (Whatman), column. The column was developed with a gradient of 1 M (NH_4_)_2_HPO_4_, pH 3.8 (pH adjusted with phosphoric acid): 0% for 5 min, 1–2% over 15 min, 2% for 80 min, 2–10% over 20 min, 10% for 65 min, 10–80% over 40 min, 80% for 20 min and finally 80-0%; flow rate, 1.0 ml/min [48]. The value of each glycerol-inositol corresponding to PtdIns3*P*, PtdIns4*P*, PtdIns5*P*, PtdIns(3,5)*P*_2_, and PtdIns(4,5)*P*_2_ is reported as percent of total phosphoinositol, to normalize number of cells and incorporation of [^3^H] inositol.

## Supporting Information

Figure S1 Semithin section analysis of the cerebellum from *Mtmr2*+/+*Fig4*−/− and *Mtmr2*−/−*Fig4*−/− mice at P8. A disorganization of both the molecular and granular layer was observed in the cerebellum of *Mtmr2*+/+*Fig4*−/− and *Mtmr2*−/−*Fig4*−/− mice. Middle panels show loss of Purkinjie and basket cells which are not aligned at the border of the granular layer. Inset is showing a cell carrying vacuoles in the granular layer. Bar is 80 µm in (A–C); 50 µm in (A′–C′); 80 µm in (A″) and 50 µm in (B″, C″).
(TIF)

Figure S2 Semithin section analysis of the cerebellum from *Mtmr2*+/+*Fig4*−/− and *Mtmr2*−/−*Fig4*−/− mice at P20. The loss of Purkinjie and basket cells is even more evident at P20 at the border of the granular layer. More vacuolated cells are present in the granular layer. Arrows indicate vacuolated cells. Bar is 80 µm in (A–C) and 50 µm in (A′–C′).
(TIF)

Figure S3 (A) Example of shRNA PIKfyve lentiviral vector titration to choose the highest amount of virus that did not significantly inhibit myelination nor affect the quality of myelination. (B) Western blot analysis of lysates from *Mtmr2*-null co-cultures transduced with the selected PIKfyve shRNA (12.5%) and scramble, and treated with either 70 nM of YM201636 inhibitor or DMSO, as control. MAG (myelin associated glycoprotein) is not decreased in YM201636 treated cultures, showing that treatment with the compound does not affect myelination. (C, D) Western blot analysis of FIG4 on a GST-pull down assay performed using recombinant GST-MTMR2 on extracts from total brain (C) and isolated rat Schwann cells (D). Dlg1 was detected in the Schwann cell lysate and used as a positive control for the GST-MTMR2 pull down. Note that Fig4 binds not specifically to GST and/or Sepharose beads. (E) Comassie gel showing the quality and the amount of recombinant GST-MTMR2 and GST control.
(TIF)

Table S1 PI measurements from wild-type yeast strains transformed with FLAG-MTMR2. Values listed are the percent of total phosphatidylinositol. Values for two independent experiments, as well as the averages (Avg) for each time point are presented.
(DOC)
