# Supplementary figures and images for: Genetic Interaction between MTMR2 and FIG4 Phospholipid Phosphatases Involved in Charcot-Marie-Tooth Neuropathies
Source: PLoS Genet. 2011 Oct 20;7(10):e1002319. doi: 10.1371/journal.pgen.1002319 (PMC3197679; doi:10.1371/journal.pgen.1002319)

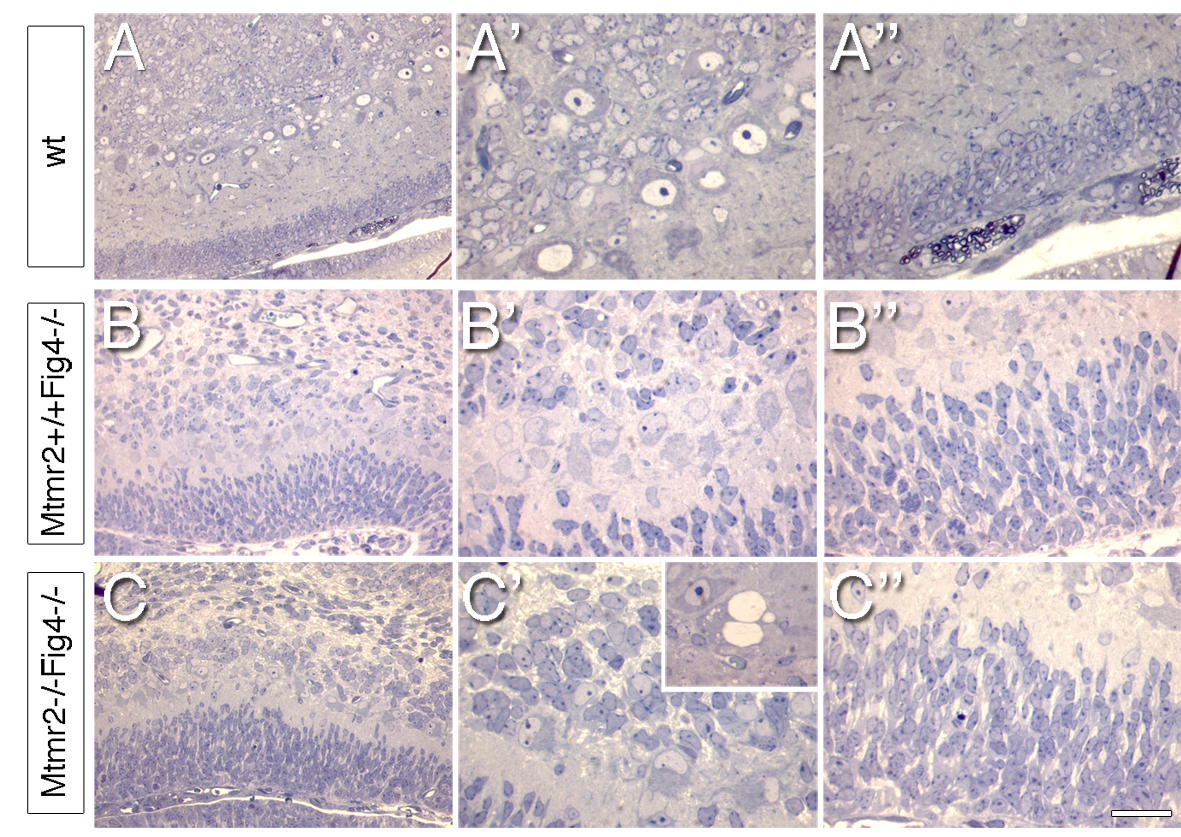

Supplement: Figure S1 — Semithin section analysis of the cerebellum from Mtmr2+/+Fig4−/− and Mtmr2−/−Fig4−/− mice at P8. A disorganization of both the molecular and granular layer was observed in the cerebellum of Mtmr2+/+Fig4−/− and Mtmr2−/−Fig4−/− mice. Middle panels show loss of Purkinjie and basket cells which are not aligned at the border of the granular layer. Inset is showing a cell carrying vacuoles in the granular layer. Bar is 80 µm in (A–C); 50 µm in (A′–C′); 80 µm in (A″) and 50 µm in (B″, C″). (TIF) [file pgen.1002319.s001.tif]

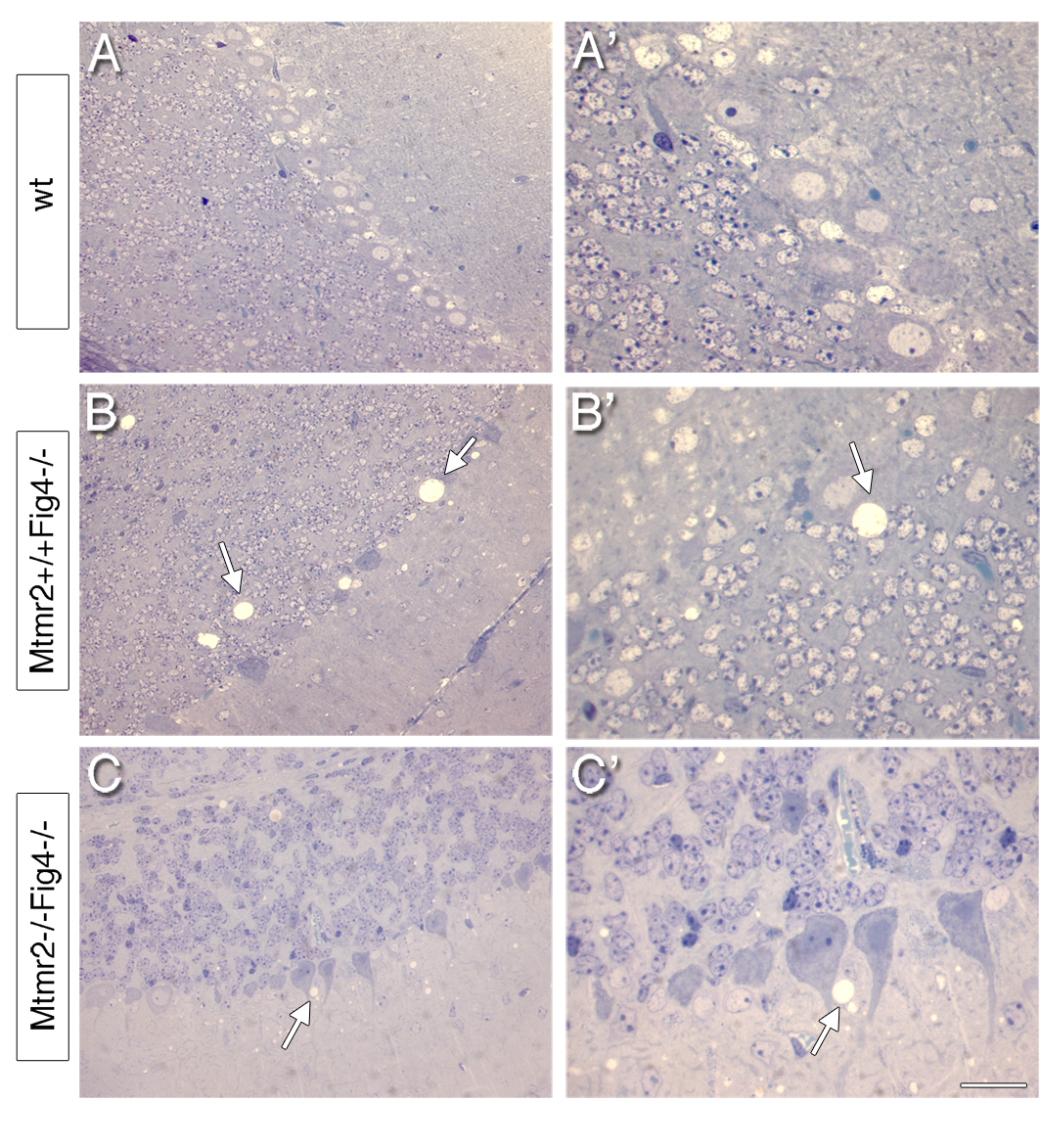

Supplement: Figure S2 — Semithin section analysis of the cerebellum from Mtmr2+/+Fig4−/− and Mtmr2−/−Fig4−/− mice at P20. The loss of Purkinjie and basket cells is even more evident at P20 at the border of the granular layer. More vacuolated cells are present in the granular layer. Arrows indicate vacuolated cells. Bar is 80 µm in (A–C) and 50 µm in (A′–C′). (TIF) [file pgen.1002319.s002.tif]

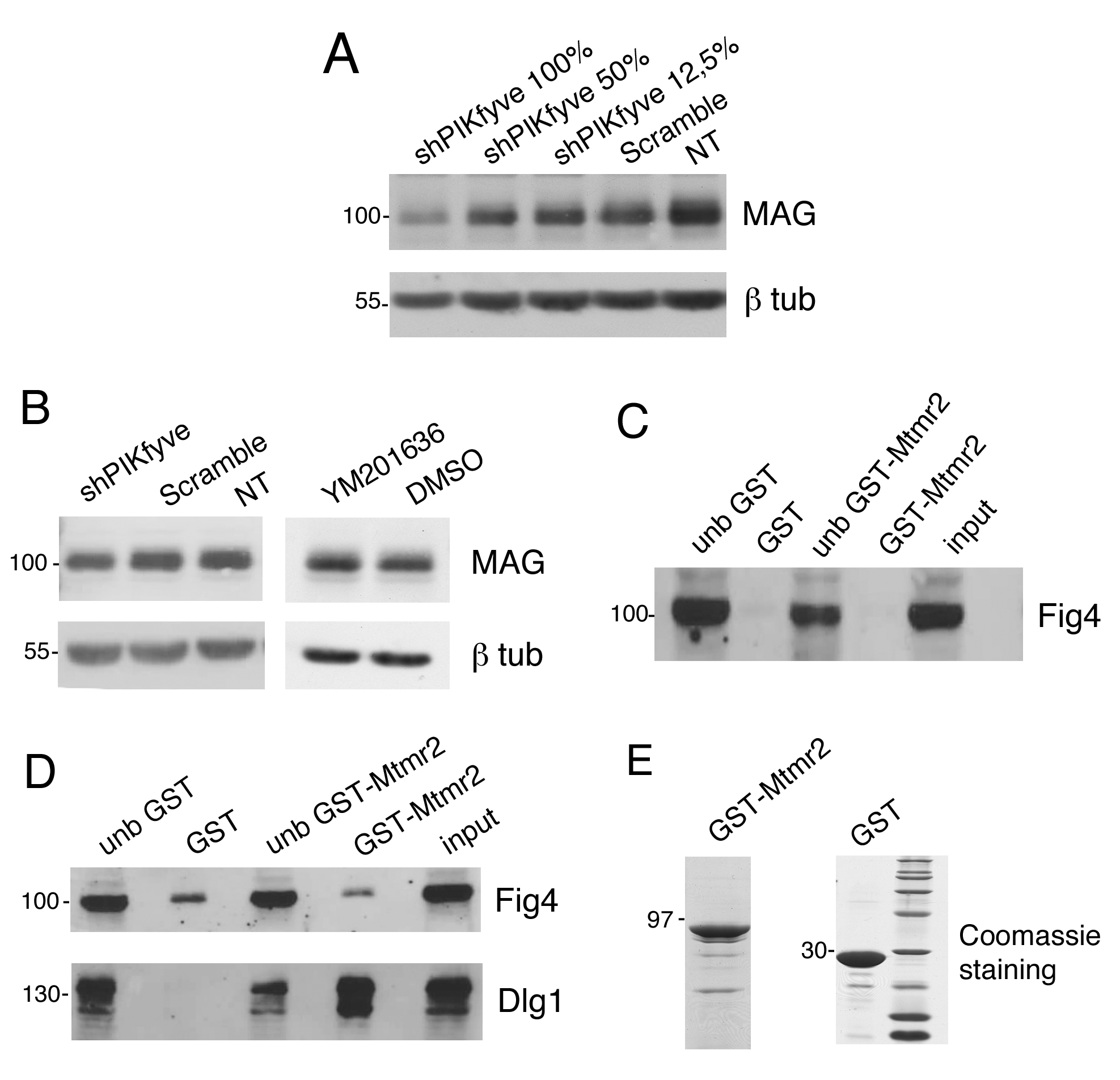

Supplement: Figure S3 — (A) Example of shRNA PIKfyve lentiviral vector titration to choose the highest amount of virus that did not significantly inhibit myelination nor affect the quality of myelination. (B) Western blot analysis of lysates from Mtmr2-null co-cultures transduced with the selected PIKfyve shRNA (12.5%) and scramble, and treated with either 70 nM of YM201636 inhibitor or DMSO, as control. MAG (myelin associated glycoprotein) is not decreased in YM201636 treated cultures, showing that treatment with the compound does not affect myelination. (C, D) Western blot analysis of FIG4 on a GST-pull down assay performed using recombinant GST-MTMR2 on extracts from total brain (C) and isolated rat Schwann cells (D). Dlg1 was detected in the Schwann cell lysate and used as a positive control for the GST-MTMR2 pull down. Note that Fig4 binds not specifically to GST and/or Sepharose beads. (E) Comassie gel showing the quality and the amount of recombinant GST-MTMR2 and GST control. (TIF) [file pgen.1002319.s003.tif]
