# Supplementary material for: Genetic Interaction between MTMR2 and FIG4 Phospholipid Phosphatases Involved in Charcot-Marie-Tooth Neuropathies
Source: PLoS Genet. 2011 Oct 20;7(10):e1002319. doi: 10.1371/journal.pgen.1002319 (PMC3197679; doi:10.1371/journal.pgen.1002319)
Supplement: Table S1 — PI measurements from wild-type yeast strains transformed with FLAG-MTMR2. Values listed are the percent of total phosphatidylinositol. Values for two independent experiments, as well as the averages (Avg) for each time point are presented. (DOC) [file pgen.1002319.s004.doc]

**Table S**1. PI measurements from wild-type yeast strains transformed with FLAG-MTMR2.

| **WT with pMTMR2** | | | | | | | | | | |
| --- | --- | --- | --- | --- | --- | --- | --- | --- | --- | --- |
|  | | 0 min | Avg | | 5 min | Avg | 10 min | Avg | 20 min | Avg |
| IP 3 | Exp 1 | 1.662 | 1.596 | 0.963 | | 1.048 | 1.074 | 1.105 | 1.098 | 1.071 |
|  | Exp 2 | 1.530 |  | 1.134 | |  | 1.136 |  | 1.045 |  |
| IP 5 | Exp 1 | 0.257 | 0.168 | 0.520 | | 0.415 | 0.541 | 0.463 | 0.328 | 0.334 |
|  | Exp 2 | 0.080 |  | 0.311 | |  | 0.385 |  | 0.340 |  |
| IP 3,5 | Exp 1 | 0.009 | 0.019 | 0.898 | | 0.923 | 0.576 | 0.469 | 0.076 | 0.061 |
|  | Exp 2 | 0.029 |  | 0.949 | |  | 0.416 |  | 0.047 |  |
| **WT with Vector** | | | | | | | | | | |
|  | | 0 min | Avg | | 5 min | Avg | 10 min | Avg | 20 min | Avg |
| IP 3 | Exp 1 | 1.875 | 2.071 | | 1.119 | 1.338 | 1.425 | 1.621 | 1.335 | 1.432 |
|  | Exp 2 | 2.267 |  |  | 1.558 |  | 1.816 |  | 1.529 |  |
| IP 5 | Exp 1 | 0.086 | 0.084 | | 0.236 | 0.171 | 0.347 | 0.245 | 0.255 | 0.282 |
|  | Exp 2 | 0.083 |  |  | 0.105 |  | 0.144 |  | 0.309 |  |
| IP 3,5 | Exp 1 | 0.033 | 0.030 | | 1.089 | 1.205 | 0.702 | 0.773 | 0.119 | 0.103 |
|  | Exp 2 | 0.027 |  |  | 1.322 |  | 0.844 |  | 0.088 |  |
